# Tumorigenesis of basal muscle invasive bladder cancer was mediated by PTEN protein degradation resulting from *SNHG1* upregulation

**DOI:** 10.1186/s13046-024-02966-4

**Published:** 2024-02-17

**Authors:** Tengda Li, Maowen Huang, Ning Sun, Xiaohui Hua, Ruifan Chen, Qipeng Xie, Shirui Huang, Mengxiang Du, Yazhen Zhao, Qianqian Lin, Jiheng Xu, Xiaoyun Han, Yunping Zhao, Zhongxian Tian, Yu Zhang, Wei Chen, Xian Shen, Chuanshu Huang

**Affiliations:** 1 Oujiang Laboratory (Zhejiang Lab for Regenerative Medicine, Vision and Brain Health), Key Laboratory of Laboratory Medicine, Ministry of Education, School of Laboratory Medicine and Life Sciences, Wenzhou Medical University, Wenzhou, Zhejiang 325035 China; 2 The First Affiliated Hospital of Wenzhou Medical University, Wenzhou, Zhejiang 325027 China

**Keywords:** lncRNA SNHG1, PTEN, USP8, Bladder cancer

## Abstract

**Background:**

Phosphatase and tensin homolog deleted on chromosome ten (PTEN) serves as a powerful tumor suppressor, and has been found to be downregulated in human bladder cancer (BC) tissues. Despite this observation, the mechanisms contributing to PTEN’s downregulation have remained elusive.

**Methods:**

We established targeted genes’ knockdown or overexpressed cell lines to explore the mechanism how it drove the malignant transformation of urothelial cells or promoted anchorageindependent growth of human basal muscle invasive BC (BMIBC) cells. The mice model was used to validate the conclusion in vivo. The important findings were also extended to human studies.

**Results:**

In this study, we discovered that mice exposed to N-butyl-N-(4-hydroxybu-tyl)nitrosamine (BBN), a specific bladder chemical carcinogen, exhibited primary BMIBC accompanied by a pronounced reduction in PTEN protein expression in vivo. Utilizing a lncRNA deep sequencing high-throughput platform, along with gain- and loss-of-function analyses, we identified small nucleolar RNA host gene 1 (*SNHG1*) as a critical lncRNA that might drive the formation of primary BMIBCs in BBN-treated mice. Cell culture results further demonstrated that BBN exposure significantly induced *SNHG1* in normal human bladder urothelial cell UROtsa. Notably, the ectopic expression of *SNHG1* alone was sufficient to induce malignant transformation in human urothelial cells, while *SNHG1* knockdown effectively inhibited anchorage-independent growth of human BMIBCs. Our detailed investigation revealed that *SNHG1* overexpression led to PTEN protein degradation through its direct interaction with HUR. This interaction reduced HUR binding to ubiquitin-specific peptidase 8 (USP8) mRNA, causing degradation of USP8 mRNA and a subsequent decrease in USP8 protein expression. The downregulation of USP8, in turn, increased PTEN polyubiquitination and degradation, culminating in cell malignant transformation and BMIBC anchorageindependent growth. In vivo studies confirmed the downregulation of PTEN and USP8, as well as their positive correlations in both BBN-treated mouse bladder urothelium and tumor tissues of bladder cancer in nude mice.

**Conclusions:**

Our findings, for the first time, demonstrate that overexpressed *SNHG1* competes with USP8 for binding to HUR. This competition attenuates USP8 mRNA stability and protein expression, leading to PTEN protein degradation, consequently, this process drives urothelial cell malignant transformation and fosters BMIBC growth and primary BMIBC formation.

**Supplementary Information:**

The online version contains supplementary material available at 10.1186/s13046-024-02966-4.

## Background

Bladder cancer (BC) ranks as the 10th most common cancer, accounting for 573,278 new cases and 212,536 new deaths in 2021 [1]. Most BC cases necessitate vigilant, lifelong monitoring and intervention, making BC the costliest cancer to manage [1–3]. Muscle invasive bladder cancer (MIBC) often portends a grim prognosis, with distantly metastatic BCs exhibiting only a 5% of 5-year survival rate [4–6]. Therefore, the exploration of potential driver oncogenes and the underlying molecular mechanisms that promote primary basal muscle invasive BC (BMIBC) development are paramount. Such discoveries could profoundly impact the identification of new carcinogenic mechanisms, biomarkers for diagnosing, and treatment of human BC patients.

Phosphatase and tensin homolog deleted on chromosome ten (PTEN), a potent tumor suppressor, frequently loses function during the cancer development process [7]. PTEN plays roles in intracellular phosphatase-(in)dependent activities [7], and governs various biological processes such as cellular genomic stability, self-renewal or metabolism balance [7–9]. Even minor alterations in PTEN expression can fuel tumor progression [7, 9, 10]. Clinical studies have revealed PTEN downregulation in many BC tissues [11], suggesting that restoring PTEN’s tumor suppressor function could forge a new pathway for BC management. As PTEN has a broad spectrum of downstream regulatory pathways without specific tendencies [7, 12–15], pinpointing the key nodes that reduce PTEN expression in BC may be vital for effective treatment.

Small nucleolar RNA host gene 1 (*SNHG1*), a long noncoding RNA (lncRNA) spanning approximately 1.6 kb and located at chromosome 11q12.3 [16, 17], has been found to be abnormally overexpressed in various cancers, including pituitary and breast cancers [18, 19]. Our investigation into bladder-specific carcinogen N-butyl-N-(4-hydroxybu-tyl)nitrosamine (BBN)-caused mouse basal MIBCs employed lncRNA deep sequencing with the Illumina HiSeqTM2000/2500 high-throughput platform to evaluate 5,442 known lncRNAs. This analysis revealed that BBN treatment modulated 17 lncRNAs in mouse urothelium compared to vehicle-treated controls. Utilizing real-time PCR and gain- and loss-function analyses, we identified *SNHG1* as a key lncRNA candidate that might drive basal MIBCs formation in BBN-treated mice. Though *SNHG1* was significantly elevated in both BBNtreated mouse urothelium and human MIBCs [20], the molecular mechanisms underlying *SNHG1*’s role in bladder carcinogenesis remain largely uncharted. In our current studies, we unveiled that overexpressed *SNHG1* could promote PTEN protein degradation, consequently driving urothelial transformation and BMIBC cell growth/tumorigenicity. This effect is mediated through the attenuation of ubiquitin-specific peptidase 8 (USP8) mRNA stability and protein expression.

## Materials and methods

### Plasmids, antibodies and reagents

#### Plasmids

The full-length *Homo sapiens SNHG1* sequences was cloned into pmR-ZsGreen1 (Takara Bio, Otsu, Shiga, Japan), and shRNA constructs targeting human *SNHG1* (sh*SNHG1*) were placed into the p-GIPZ vector. The USP8 promoter was incorporated into the pGL3 basic luciferase reporter. Enhanced FLAG/HA-USP8, shRNA constructs for USP8 or nucleolin (NCL), HA-MS2 overexpression vector, pSL-MS2-12X plasmids, and PTEN mRNA 3’-UTR luciferase reporter were constructed as described in previous studies [21–24]. Additionally, the *SNHG1*-MS2-overexpressing plasmid was constructed into pSL-MS2-12X vector.

#### Antibodies

Various specific antibodies were procured from reputable sources (ELAV like RNA binding protein 1, also known as ELVAL1 (HUR) and NCL): Santa Cruz Biotechnology (Santa Cruz, CA, USA); PTEN, PP2A-A, PP2A-B, PP2A-C, NEDD4, FBW7, ITCH, USP4, HA or Flag: Cell Signaling Technology (Beverly, MA, USA); PHLPP1 and PHLPP2: Bethyl Laboratories (Montgomery, TX, USA); p63α: Genetex (Irvine, CA, USA); XIAP: BD Biosciences (San Jose, CA, USA); Anti-AUF1: Aviva (San Diego, CA, USA); USP8, β-Actin and GAPDH: Proteintech (Rosemont, IL, USA).

#### Reagents

Actinomycin D (Act D): Santa Cruz (Dallas, TX, USA); BBN (B0938): TCI American (Portland, OR, USA); MG132 and protein synthesis inhibitor cyclohexamide (CHX): Calbiochem (San Diego, CA, USA).

##### Cell culture and transfection

Human normal bladder urothelial cell line UROtsa was employed in accordance with our earlier publications [25]. Additionally, the human bladder cancer cell line U5637, kindly provided by Dr. Xue-Ru Wu of the Departments of Urology at New York University School of Medicine, was utilized in this study. Both cell lines were cultured at 37 °C in a 5% CO_2_ incubator using RPMI 1640 medium supplemented with 10% fetal bovine serum (FBS) and 2 mM L-glutamine. The authenticity of all cell lines was verified before/after usage in the research by Genetica DNA Laboratories, utilizing the PowerPlex® 16 HS System (Burlington, NC, USA). Transfections were executed using PolyJet™ DNA in vitro transfection reagent (SignaGen Laboratories, Rockville, MD, USA), in line with the manufacturer’s guidelines. Selection of stable transfectants was performed using puromycin, hygromycin B or G418 (Life Technologies), depending on the constructs, for a period of 4–6 weeks. Surviving cells post-antibiotics selection were pooled to form stable mass transfectants.

##### Western blot

For the experiments conducted in this study, UROtsa cells and U5637 cells, along with their transfectants, were synchronized by being cultured in 0.1% FBS 1640 medium for 12 h. Subsequently, they were cultured in 10% FBS medium for an additional 12 h prior to collection for further analysis, unless otherwise specified. UROtsa cells underwent treatment with BBN (400 μM) for various durations as indicated in the study. Cells extracts were prepared using cell lysis buffer, and protein concentrations were ascertained prior to performing Western blots, as detailed in our previous studies [21]. The resulting images were captured utilizing the PhosphorImager Typhoon FLA 7000 imager (GE Healthcare, Pittsburgh, PA).

##### RNA immunoprecipitation (RIP) assay

The RIP assay was carried out following the protocol delineated in our earlier publications [21]. To summarize, approximately 1 × 10^7^ cells were collected and treated with polysome lysis buffer. Subsequently, the cell lysates were centrifuged at 14000 g for 10 min at 4 °C. Antibodies against HUR or control IgG (Santa Cruz, CA, USA), along with protein Gagarose (Santa Cruz, CA, USA), were added to the supernatants and incubated overnight at 4 °C in NET2 buffer (50 mM Tris–HCl, pH7.4, 150 mM sodium chloride, 1 mM magnesium chloride, 0.05% IGEPAL, 50 U/mL RNase OUT, 50U/mL Superase IN, 1 mM dithiothreitol, and 30 mM EDTA.

Following three washes, the beads were resuspended in 100 *μ*L NET2 and 100 *μ*L SDS-TE (20 mM Tris–HCl, pH 7.5, 2 mM EDTA, and 2% sodium dodecyl sulfate) and then incubated at 55 °C for 30 min, with occasional mixing. The RNAs within the bead mixture were then extracted using miRNeasy Mini Kit (QIAGEN, Valencia, CA, USA), and quantitative real-time PCR was conducted to identify the specific RNAs within the immune-complex as previously detailed [23].

##### Immunoprecipitation assay

U5637 cells were cultured in 10-cm dishes until they reached 80–85% confluence. Following this, the cells were transiently transfected with the indicated plasmids. As per our previous study [26], 36 h post-transfection, the cells were lysed in 1 × cell lysis buffer (Cell Signaling Technology, Beverly, MA, USA) on ice. The cell lysate (500 mg) was incubated with a specific antibody and protein G-agarose beads at 4 °C overnight. The beads were washed 4–5 times with 1 × cell lysis buffer before being processed for Western blot assays.

##### Assay of cell proliferation

Cells were inoculated into 96-well plates and cultured until they achieved 70% confluence. The cells underwent an initial incubation in a medium supplemented with 0.1% FBS for 12 h, followed by further incubation in a medium containing 10% FBS for a specified duration. Post-cultivation, the supernatant in the 96-well plates was aspirated, and each well was supplemented with an equilibrated volume of ATP reagent and PBS. This was homogenized using an orbital shaker and allowed a quiescent period of 10 min for adequate mixing. The resultant luminescence was quantified using a Centro LB 960 luminometer (Berthold Technologies, Bad Wildbad, Germany). The Relative Proliferation Index was defined as the relative ATP values derived using the ATP measurement of cells harvested on the first day of culture.

##### Anchorage-independent growth assay

The experimental protocol of anchorage-independent growth commenced with 0.5% of the acrylamide lower gel layer, followed by the overlay of 0.33% upper gel mixed with quantitatively adjusted cells. The plates were incubated at 37 °C in a humidified atmosphere with 5% CO_2_. Microscopic examination and documentation of clonal expansion in sixwell plates were performed, with emphasis on quantifying and analyzing clones exceeding 32 cells in size. Detailed steps can be found in our previous research [25].

##### Determination of cell cycle

Cells were seeded into six-well plates and cultured till around 40% confluence, and then cultured sequentially in media containing 0.1% FBS for 36 h and then followed by 10% FBS for 12 h. The cells were then trypsinized, transferred into EP tubes, and fixed overnight at 4 °C in 70% ethanol. A solution of 40 μL RNase A and 360 μL Propidium Iodide (PI) was added and the samples were incubated at ambient temperature for 30 min. Finally, cellular analysis was conducted using flow cytometry on a CytoFLEX platform (Beckman Coulter, San Diego, California, USA). Detailed methodologies of these experimental procedures are comprehensively described in our previously published studies [27, 28].

##### Xenograft tumorigenic model in vivo nude mice

The xenograft tumor experiments were conducted at the Animal Institute of Wenzhou Medical University, in compliance with the protocols sanctioned by the Medical Experimental Animal Care Commission of Wenzhou Medical University. A total of sixteen female athymic nude mice, aged 3–4 weeks, were obtained from Shanghai Silaike Experimental Animal Company (license no. SCXK, Shanghai 20100002). At 5–6 weeks of age, the mice were randomly divided into two distinct groups as designated and then subcutaneously injected with various BC cell transfectants (5 × 10^6^ suspended in 100 *μ*L PBS) in the axillary region. These mice were maintained in a sterile environment and were in accordance with the guidelines of the American Association for the Accreditation of Laboratory Animal Care. The development and size of tumors were monitored and measured twice a week using calipers, with the volume calculated using the formula: volume (mm^3^) = (width^2^ [mm^2^] × length [mm])/2. Four weeks’ post-injection, the mice were euthanized, and the tumors were surgically excised, photographed and weighed. There were no premature deaths or required sacrifices before the conclusion of the in vivo study.

##### Quantitative real-time PCR

Total RNA was isolated utilizing TRIzol reagent (Invitrogen, Carlsbad, CA, USA), following the manufacturer’s guidelines. Subsequent cDNA synthesis was carried out with a Thermo-Script RT-PCR system (Invitrogen, Carlsbad, CA, USA). PCR amplification was executed using PowerUp SYBR Green Master Mix (Invitrogen, Grand Island, NY, USA), in conjunction with specific primers, as detailed in Table 1.

**Table 1:** Specific primers used for PCR amplification

	Forward	Reverse
Human *SNHG1*	5’-AGCAGACACAGATTAAGACA-3’	5’-GGCAGGTAGATTCCAGATAA-3’
Human USP8	5′-GGTTCTGGACCAGCTCTTAC-3′	5′-CTGCCACTTCACCTTTATGC-3′
Human PTEN	5′-ACACCGCCA AATTTA ACTGC -3′	5′-TACACCAGTCCGTCCCTTTC-3′
Human GAPDH	5’-GATGATCTTGAGGCTGTTGTC-3’	5’-CAGGGCTGCTTTTAACTCTG-3’

##### Luciferase reporter assay

The assessment of luciferase activity was carried out using a luciferase assay system kit (Promega Corp., Madison, WI, USA), in alignment with previously described methods [29]. Cells were transiently co-transfected with pRL-TK together and either the associated promoter-driven luciferase reporter or mRNA 3’-UTR luciferase reporter. 24 h’ post-transfection, cell extracts were prepared and subjected to the luciferase assay using the designated kit. The luciferase activity was normalized against the internal TK signal, and the outcomes are presented as the mean ± SE from triplicates experiments.

##### Experiments for mouse exposed to BBN in drinking water

The C57BL/6 mice, aged 6 to 8 weeks, were randomly allocated into two main groups: a vehicle-treated control group and a group treated with BBN [22, 30]. Mice in BBN-treated group were administered BBN (0.05%) in their drinking water for the specified time periods, while the control group received regular drinking water. Upon completion of the treatment, the mice were euthanized, and their bladder were surgically removed, photographed, and dissected. A portion of each bladder was fixed overnight in 4% paraformaldehyde at 4 °C, then processed for paraffin embedding, followed by hematoxylin and eosin (H & E) staining. The remaining bladder tissue was used to harvest mouse urothelium, subsequently evaluated for *SNHG1* expression levels using Real-time PCR.

##### Immunohistochemistry Staining (IHC)

Bladder tissues from the mice were immunostained with specific antibodies targeting PTEN (Beverly, MA, USA) and USP8 (Rosemont, IL, USA). The resulting immunostaining images were captured utilizing the AxioVision Rel.4.6 computerized image analysis system (Carl Zeiss, Oberkochen, Germany). Protein expression levels were quantified by calculating the integrated optical density per stained area (IOD/area) with Image-Pro Plus version 6.0 (Media Cybernetics, MD, USA). The IHC-stained sections were evaluated at 400-fold magnifications, and at least 5 representative fields from each section were analyzed to calculate the optical density, based on characteristic photographs.

##### Statistical methods

All statistical analysis was conducted using GraphPad Prism 5.0. Student’s *t*-test was applied to determine the significance between different groups, while the correlation between two continuous variables was assessed using Pearson coefficient. Data are presented as the means ± standard deviation (SD), and the Student’s *t*-test was employed to determine the *p*-value when comparing two continuous datasets. A *p*-value of less than 0.05 was considered statistically significant.

## Results

### BBN treatment resulted in primary BMIBC formation concurrent with remarkably induction of* SNHG1 *in vivo*,* while overexpressed *SNHG1* promoted tumorigenicity of human BMIBCs in *nude mice*

**Fig. 1:**
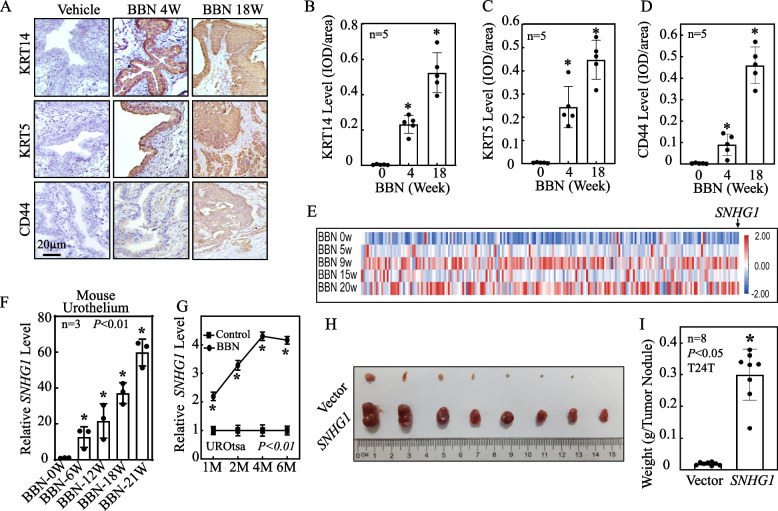
BBN treatment led to the primary formation of BMIBC, coinciding with a remarkable induction of *SNHG1 *in vivo. Concurrently, the overexpression of *SNHG1* was observed to enhance tumorigenicity in nude mice. **A**-**D** BBN treatment induced protein alterations of KRT14, KRT5 and CD44 in vivo. The protein levels were quantitated and presented by IOD/area. **E** A heat map illustrated the high-throughput sequencing results of BBN mouse urothelium at varying stages. **F** Mice in the BBN-treated group were administered BBN (0.05%) treatments through their drinking water as indicated, while the negative control group was given normal drinking water. The mice were sacrificed at specific time points, and their bladders were surgically removed to collect mouse urothelium. Real-time PCR was employed to assess *SNHG1* expression levels. **G** Human normal bladder urothelial cell line UROtsa cells were treated with 400 μM BBN for different times as indicated. The cells were then extracted for total RNA isolation with TRIzol reagent and real-time PCR was performed to determine *SNHG1* expression levels. GAPDH was used as an internal control. M, indicates month. **H**-**I** Athymic nude mice were subcutaneously injected in the right axillary region with *SNHG1*overexpressed cells and corresponding vector scramble transfectants (5 × 10^6^ suspended in 100 *μ*L PBS). Six weeks’ post-injection, the mice were sacrificed, and the tumors were surgically excised and photographed (**H**), as well as weighed (**I**). An asterisk (*) denotes a significant increase compared to Vector transfectants (*p* < 0.05)

BBN is a specific carcinogen for bladder cancer. When mice were exposed to BBN in their drinking water for over 18 weeks, there was a 100% (12/12) incidence rate of high-grade, muscleinvasive BCs [30]. Our data showed that extended exposure to BBN led to a gradual increase in the expression of BMIBC biomarkers–namely KRT14, KRT5, and CD44 in the urothelium of mouse bladders (Fig. 1A-D). After 18 weeks of BBN treatment, the mice essentially developed primary BMIBC (Fig. 1A-D). The results obtained from using the Illumina HiSeqTM2000/2500 highthroughput platform to evaluate 5442 known lncRNAs in BBN-treated mouse urothelium revealed that BBN treatment for 20 weeks (*n* = 10) either up- or down-regulated 17 lncRNAs in mouse urothelium as compared with vehicle-treated (*n* = 10) mice (Fig. 1E, Table S1). We have uploaded the raw sequencing data to the shared platform Github. The detailed link is https://github.com/tengdali90/BBN-induced-bladder-cancer/issues/1. Furthermore, real-time PCR analysis demonstrated a timedependent increase in *SNHG1* expression in the mouse bladder urothelium upon BBN exposure (Fig. 1F). Similar trends were observed in The Cancer Genome Atlas (TCGA) database, which showed elevated *SNHG1* expression in patients with bladder cancer when compared to normal bladder tissues (Fig. S1A and B). We also investigated *SNHG1* expression in normal human bladder urothelial cell (UROtsa) and various human bladder cancer cell lines such as J82, UMUC3, T24T, U5637. Notably, *SNHG1* level was significantly increased in J82, UMUC3, T24T, U5637 in comparison to that in UROtsa cell line (Fig. S2). Consistent with the effects of BBN observed in mouse urothelium in vivo and human BC patients, exposure of UROtsa cells to BBN also caused a time-dependent *SNHG1* induction in comparison to these in vehicle-treated cells (Fig. 1G).

To determine the potent oncogenic activity of *SNHG1 *in vivo, *SNHG1* overexpressed and its Vector scramble transfectants were subcutaneously injected into nude mice. The ectopic expression of *SNHG1* remarkably accelerated xenograft tumor growth, increasing the tumor burden (weight) by over 658% compared to the Vector transfectants (*p* < 0.05, *n* = 8) (Figs. 1H-I, S3A).

Immunohistochemical staining further revealed a significantly upregulation of the proliferation marker Ki67 in *SNHG1*-overexpressing tumor tissues, confirming that *SNHG1* overexpression enhances BMIBC tumorigenicity in vivo (Fig. S3B-C).

### Ectopic expression of *SNHG1* alone was sufficient to drive the malignant transformation of normal human urothelial cells and to enhance the anchorage-independent growth capabilities of BMIBC cell lines

**Fig. 2:**
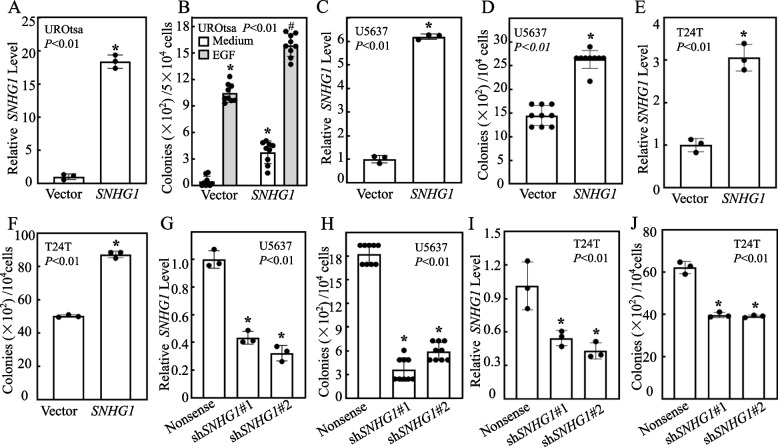
Ectopic expression of *SNHG1* alone was sufficient to drive to the malignant transformation of normal human bladder urothelial cell and to enhance the anchorageindependent growth of human BMIBC cell lines. **A**, **C**, **E** UROtsa (**A**), U5637 (**C**) and T24T (**E**) cells were stably transfected with a plasmid constitutively expressing human *SNHG1* and real-time PCR was performed to identify the stable transfectants. **B**, **D**, **F** UROtsa(*SNHG1*) and UROtsa(Vector) (**B**), U5637(*SNHG1*) and U5637(Vector) (**D**), T24T(*SNHG1*) and T24T(Vector) (**F**) cells underwent an anchorage-independent assay, the number of colonies was counted, and the results were presented as colonies per 50,000 or 10,000 cells, with bars representing the mean ± SD. In (**B**), An asterisk (*) indicates a significant increase compared to the medium control in UROtsa(Vector) cells, and the symbol (#) denotes a significant increase compared to UROtsa(Vector) cells treated with EGF (*p* < 0.01). In (**A**, **C**-**F**), an asterisk (*) signifies a significant increase compared to cells transfected with Vector. **G** and **I** Cell extracts from various U5637 (**G**) or T24T (**I**) transfectants were analyzed by real-time PCR to identify the knockdown efficiency of shRNA targeting *SNHG1*. **H** and **J** U5637(sh*SNHG1*#1) cells, U5637(sh*SNHG1*#2) cells *versus* U5637(Nonsense) cells (**H**), T24T(sh*SNHG1*#1) cells, T24T(sh*SNHG1*#2) cells *versus* T24T(Nonsense) cells (**J**) were subjected to an anchorage-independent soft agar growth assay as detailed in the Materials and Methods section. The number of colonies, each with more than 32 cells, was counted, and the results were presented as colonies/10^4^ cells. Bars show the mean ± SD, and an asterisk (*) signifies a significant decrease compared to UROtsa(Nonsense) transfectants or T24T(Nonsense) transfectants (*p* < 0.01)

Our previous studies indicated that UROtsa cells repeatedly exposed to BBN for over 6 months gain the capability of anchorage-independent growth in soft agar, indicating malignant transformation of human urothelial cells [31]. Tumorigenic ability of transformed UROtsa cells in vivo animal models had also been reported [32]. To elucidate the functional role of *SNHG1* in this transformation, we stably transfected *SNHG1* expressing construct into UROtsa cells. As evidenced in Figs. 2A-B and S4A, the ectopic expression of *SNHG1* alone was sufficient to induce malignant transformation of UROtsa cells, suggesting that *SNHG1* induction by BBN plays a critical role in driving the malignant transformation of urothelial cell. Moreover, *SNHG1* also augmented EGF-induced urothelial cell transformation (Figs. 2B and S4A). Consistently, targeted *SNHG1* knockdown in human BMIBC U5637 or T24T cells dramatically inhibited their anchorage-independent growth, while forced *SNHG1* expression substantially enhanced this capability (Figs. 2C-J, S4B-E). Our results also revealed that *SNHG1* overexpression accelerated the cell proliferation and cell cycle progression of UROtsa and T24T cells (Fig. S5A-D), while its downregulation led to cell cycle arrest in T24T cells (Fig. S5E-F). Taken together, these results underscore that *SNHG1* not only serves as a driving force for the malignant transformation of UROtsa cells, but also fosters anchorage-independent growth in human BMIBC cells in vitro.

### *SNHG1* overexpression led to PTEN protein downregulation, in turn facilitated urothelial cell transformation and BMIBC anchorage-independent growth

**Fig. 3:**
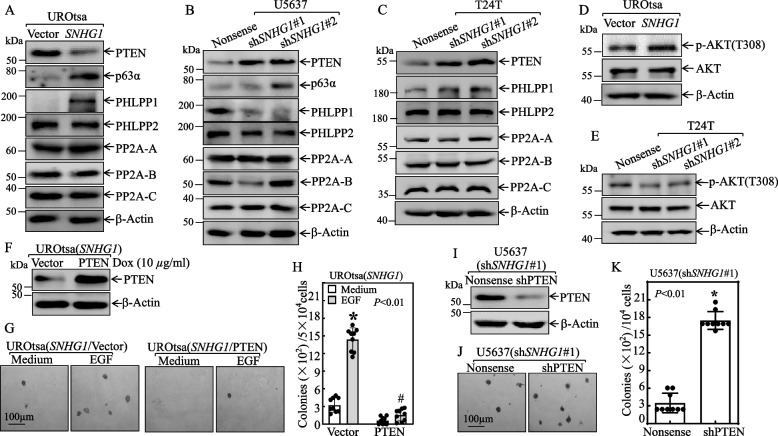
The downregulation of PTEN mediated *SNHG1’s* promotion of urothelial cell transformation, anchorage-independent cell growth and tumorigenesis in BC. **A**-**C** The indicated cell extracts were analyzed by Western blot to determine the expression of PTEN, PHLPP1, PHPPL2, p63α, PP2A-A, PP2A-B, and PP2A-C. β-Actin served as a protein loading control. **D**-**E** AKT and p-AKT (T308) were determined by Western blot in UROtsa(*SNHG1*) *versus* UROtsa(Vector) cells (**D**), and T24T(sh*SNHG1*#1) cells, T24T(sh*SNHG1*#2) cells *versus* T24T(Nonsense) cells (**E**). β-Actin was the protein loading control. **F** A PTEN-overexpressing construct was stably transfected into UROtsa(*SNHG1*) cells, and Western blot analysis was performed to confirm PTEN protein expression. **G** and **H** UROtsa(*SNHG1*/PTEN) and UROtsa(*SNHG1*/Vector) transfectants were analyzed for anchorage-independent growth in the presence or absence of EGF, as indicated. Representative images of the colonies were captured under microscopy after a 3-week incubation period. The colony count was conducted under microscopy, with results presented as colonies per 50,000 cells. An asterisk (*) signifies a significant increase compared to the medium control, while the symbol (#) denotes significant inhibition compared to UROtsa(*SNHG1*/Vector) cells treated with EGF (*p* < 0.01). **I** PTEN shRNA construct was stably transfected into U5637(sh*SNHG1*#1) cells, and PTEN knockdown efficiency was verified by Western blotting. **J** and **K** U5637(sh*SNHG1*#1/shPTEN) cells *versus* U5637(sh*SNHG1*#1/Nonsense) cells were subjected to an anchorage-independent soft agar assay. The number of colonies, each with more than 32 cells, was counted, and the results were presented as colonies/10^4^ cells. Bars depict the mean ± SD, and an asterisk (*) indicates a significant increase compared to the U5637(sh*SNHG1*#1/Nonsense) transfectant (*p* < 0.01)

In light of the existing reports that p63α, PTEN, PHLPP1 and PHLPP2 are key regulators of cell transformation and cancer cell proliferation [7, 14, 25, 33–35], we investigated the expression levels of these proteins in UROtsa(*SNHG1*) *vs.* UROtsa(Vector) cells and in U5637(sh*SNHG1*) *vs.* U5637(Nonsense) cells and in T24T(sh*SNHG1*) *vs.* T24T(Nonsense) cells. As shown in Fig. 3A, *SNHG1* overexpression in UROtsa cells led to a markedly attenuation of PTEN protein levels without affecting other proteins such as PHLPP2, PP2A-A, PP2A-B, or PP2A-C. Although induction of p63α and PHLPP1 was noticeable in UROtsa(*SNHG1*) cells (Fig. 3A), their alterations did not consistent with *SNHG1*-mediated promotion of urothelial transformation. Consistently, knockdown of *SNHG1*, elevated PTEN expression levels in U5637 or T24T cells (Fig. 3B-C). Phosphorylation of AKT at T308 (p-AKT (T308)) can be an indicator of loss of PTEN activity [36]. Our results showed that p-AKT (T308) was increased in UROtsa(*SNHG1*) *vs.* UROtsa(Vector) cells, while it was decreased in T24T(sh*SNHG1*) *vs.* T24T(Nonsense) cells, without altering the overall AKT protein levels (Fig. 3D-E). The data based on TCGA showed that PTEN protein level was higher in T1 ~ 2 *vs.* T3 ~ 4 or M0 *vs.* M1 categories in patients with bladder cancer (Fig. S1C and D). Those results indicate that *SNHG1* overexpression selectively attenuates PTEN protein expression in BCs, suggesting that PTEN attenuation might contribute to *SNHG1*-induced UROtsa cell transformation.

To address this notion, we stably transfected inducible PTEN expression construct into UROtsa(*SNHG1*) cells. These stable transfectants were verified by Western blot upon Dox treatment as shown in Fig. 3F. Ectopic expression of PTEN abolished anchorage-independent growth promoted by either *SNHG1* alone or *SNHG1* plus EGF (Fig. 3G and H). We also transfected a PTEN overexpressing construct and its corresponding vector into T24T(*SNHG1*) or T24T(Vector) cells. The results indicate that heightened PTEN levels curtail the anchorage-independent growth of T24T(*SNHG1*) or T24T(Vector) cells (Fig. S6A-C). Moreover, we transfected shRNA specific targeting PTEN construct into U5637(sh*SNHG1*#1) or T24T (sh*SNHG1*#1) cells to knockdown of PTEN expression as shown in Figs. 3I and S6D. In comparison to the U5637(sh*SNHG1*#1/Nonsense) cells or T24T(sh*SNHG1*#1/Nonsense), PTEN knockdown restored the anchorage-independent growth of U5637(sh*SNHG1*#1) cells or T24T(sh*SNHG1*#1) cells (Figs. 3J-K, S6E-F). Collectively, our results substantiate that PTEN functions as a downstream effector in the *SNHG1* signaling pathway, influencing both urothelial cell transformation and anchorageindependent growth in BMIBC cells.

### Overexpressed* SNHG1* promoted PTEN protein degradation

**Fig. 4:**
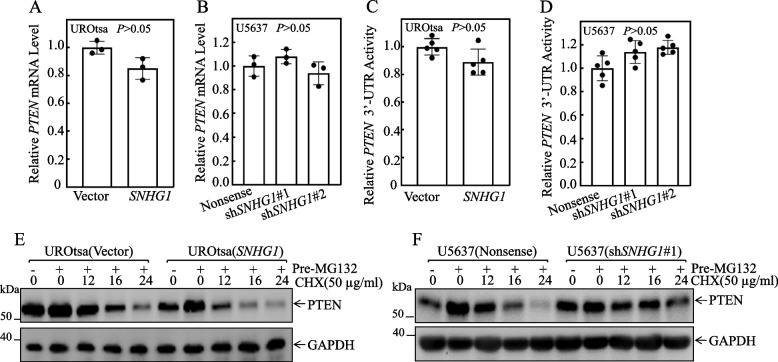
*SNHG1* promoted PTEN protein degradation. **A** and **B** Total RNA was extracted from the indicated UROtsa or U5637 transfectants using Trizol reagent. Real-Time PCR assays were then conducted to evaluate PTEN mRNA expression levels, utilizing GAPDH as an internal control. **C** and **D** The pMIR-PTEN 3’-UTR mRNA reporter was transiently transfected into the specified cells, followed by an assessment of the luciferase activity for each transfectant. The activity is depicted relative to scramble control transfectants and further normalized using an internal control, pRL-TK. Bars represent the mean ± SD from three separate experiments. **E** and **F** The designated cells were pretreated with the proteasome inhibitor MG132 (5 μM) for 10 h. Following this, the cells were exposed to CHX for the stipulated durations, and the cell extracts were subsequently analyzed through Western blotting. GAPDH served as a protein loading control

To delve deeper into the molecular mechanisms responsible for *SNHG1* downregulation of PTEN expression, we initially assessed *PTEN* mRNA levels in UROtsa cells stably transfected with *SNHG1* overexpressing construct, and compared them to cells transfected with its vector control. Figure 4A reveals no discernible differences in *PTEN* mRNA levels between UROtsa(*SNHG1*) and UROtsa(Vector) transfectants. This finding was also observed in U5637(sh*SNHG1*) *vs.* U5637(Nonsense) cells (Fig. 4B), indicating that *SNHG1*-mediated PTEN protein downregulation is beyond the mRNA level. We next determined the potential involvement of the *PTEN* mRNA 3’-UTR in *SNHG1* regulation of PTEN protein expression. *PTEN* mRNA 3’-UTR luciferase reporter was transfected into UROtsa(Vector) and UROtsa(*SNHG1*) cells. The results showed that *PTEN* mRNA 3’UTR-driven luciferase activities were comparable between UROtsa(*SNHG1*) and UROtsa(Vector) cells or in U5637(sh*SNHG1*) cells in comparison to U5637(Nonsense) cells (Fig. 4C and D)*.*

Based on the observations above, we investigated the influence of *SNHG1* overexpression on PTEN protein degradation rate. As shown in Fig. 4E, we pre-treated UROtsa(Vector) and UROtsa(*SNHG1*) cells with MG132 for PTEN protein accumulation and then the protein synthesis inhibitor CHX was applied to the cells over a following experimental time course to investigate PTEN protein degradation rates in *SNHG1* overexpressed cells in comparison to scramble vector transfectants. The results revealed that *SNHG1* overexpression remarkably accelerated PTEN protein degradation rate as compared to that observed in UROtsa(Vector), suggesting that *SNHG1* promotes PTEN protein degradation in UROtsa cells. Corroborating this, knockdown of *SNHG1* in U5637 cells profoundly reduced PTEN protein degradation rate in comparison to that U5637(Nonsense) cells (Fig. 4F), greatly supporting that overexpressed *SNHG1* promotes PTEN protein degradation.

### Overexpressed* SNHG1* promoted PTEN protein degradation through attenuation of USP8 expression

**Fig. 5:**
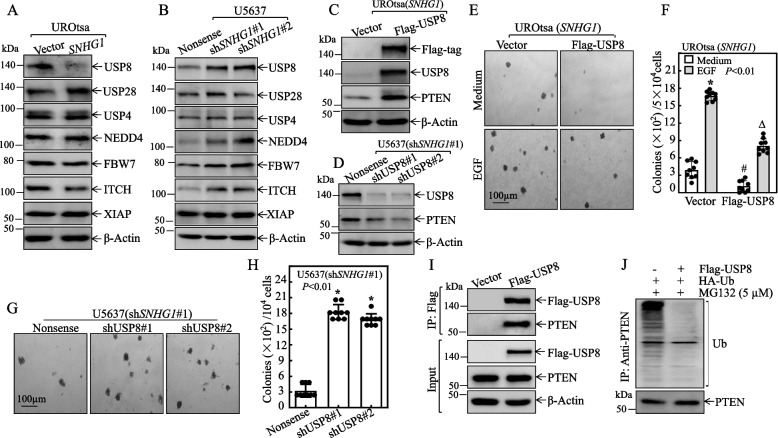
*SNHG1* attenuated USP8 expression, consequently facilitating PTEN protein degradation. **A**-**D** The selected cell extracts underwent Western blot analysis to determine the expression levels of the indicated proteins. β-Actin was used as a protein loading control. **E** and **F** UROtsa(*SNHG1*/Flag-USP8) and UROtsa(*SNHG1*/Vector) cells were evaluated for anchorage-independent growth, both with and without EGF, as specified. Following a 3-week incubation period, representative images of the colonies were captured under microscopy, and the colony count was conducted, with results presented as colonies per 50,000 cells. An asterisk (*) signifies a significant increase compared to the medium control, and the symbol (#) denotes significant inhibition relative to UROtsa(*SNHG1*/Vector) cells cultured in normal medium (*p* < 0.05), the symbol. (∆) indicates a significant decrease compared with UROtsa(*SNHG1*/Vector) cells treated with EGF (*p* < 0.05). **G** and **H** U5637 cells, including variants U5637(sh*SNHG1*#1/shUSP8#1) and U5637(sh*SNHG1*#1/shUSP8#2), were subjected to an anchorage-independent growth assay. The number of colonies, each containing more than 32 cells, was tallied, and results were depicted as colonies per 10^4^ cells. Bars represent the mean ± SD. **I** U5637 cells were transfected with Flag-USP8 or its vector control constructs, followed by coimmunoprecipitation with anti-Flag antibody-conjugated agarose beads. The immunoprecipitates were subsequently analyzed through immunoblotting to detect PTEN. **J** U5637 cells were transfected with ubiquitin-WT constructs in conjunction with Flag-USP8, as indicated. Transfectants were treated with the proteasome inhibitor MG132 (5 μM) for 10 h, 24 h’ post-transfection. The cells were then lysed and co-immunoprecipitated with anti-PTEN antibody-conjugated agarose beads, followed by Western blot analysis using anti-Ub to detect PTEN ubiquitination

Ubiquitination and de-ubiquitination are well-established mechanisms governing protein degradation across a variety of cancers [37, 38]. Specifically, the ubiquitination process involves a sequential cascade of E1, E2, and E3 enzymes, while de-ubiquitinating enzymes can reverse this modification by removing ubiquitin chains from targeted proteins [37, 39, 40]. Prominent E3 ubiquitin ligases include NEDD4, XIAP, FBW7 and ITCH, while USP8, USP4, USP28 are among a family of over 70 deubiquitinases (DUBs) present in humans and other mammals [37, 39, 41]. PTEN protein degradation was recently reported to be mediated by the E3 ubiquitin ligase NEDD4 [42]. To determine the *SNHG1* downstream effectors that are responsible for promoting PTEN protein degradation, we evaluated the effect of *SNHG1* expression on NEDD4, XIAP, FBW7, ITCH, USP8, USP4, USP28 in UROtsa(*SNHG1*) *vs.* UROtsa(Vector) and in U5637(sh*SNHG1*) *vs.* U5637(Nonsense) cells. As shown in Fig. 5A, overexpression of *SNHG1* specifically inhibited USP8 expression with no remarkable effect on the expression of other potential E3 ubiquitin ligases or Ubiquitin specific peptidases in UROtsa cells. Consistently, the impaired expression of *SNHG1* by its specific shRNA resulted in a dramatic increase in USP8, NEDD4 and ITCH, and no significant impact on expression of XIAP, FBW7, USP4 and USP28 in U5637 cells (Fig. 5B).

Considering the influencing of *SNHG1* on expression of these protein expressions and their biological functions, we hypothesized that USP8 could be a downstream effector of *SNHG1,* mediating PTEN protein degradation. To validate this notion, we stably transfected Flag-USP8 into UROtsa(*SNHG1*), while shRNAs specifically targeting USP8 (shUSP8#1 and shUSP8#2) were stably introduced into U5637(sh*SNHG1*#1). Our data demonstrated that ectopic expression of Flag-USP8 restored PTEN levels in UROtsa(*SNHG1*) cells, while USP8 knockdown attenuated PTEN levels in U5637(sh*SNHG1*#1) cells (Fig. 5C and D). This strongly suggests that USP8 is pivotal in the *SNHG1*driven degradation of PTEN protein. Consistent with positive regulatory effect of USP8 on PTEN expression, ectopic expression of USP8 impaired the cell transformation due to *SNHG1* overexpression (Fig. 5E and F), while knockdown of USP8 in U5637(sh*SNHG1)* cells profoundly rescued the anchorage-independent growth ability of U5637(sh*SNHG1*) cells (Fig. 5G and H). Further supporting these findings, USP8 knockdown in T24T(sh*SNHG1*#1) led to a decrease in PTEN protein expression and an enhancement of their anchorage-independent growth capabilities (Fig. S7A-C). Collectively, our results strongly indicate that USP8 serves as a critical mediator in the oncogenic effect of *SNHG1* on human urothelial transformation and BC anchorage-independent growth by facilitating PTEN protein degradation. To explore whether PTEN protein might be a direct substrate of USP8, given its role as a de-ubiquitinating enzyme, we performed an immunoprecipitation assay. As shown in Fig. 5I, PTEN was presented in the immune-complex pulled down of Flag-USP8 using anti-USP8 antibodies, strongly indicating the interaction USP8 enzyme with PTEN protein. This was further corroborated by evidence showing that USP8 overexpression eliminated the ubiquitination of PTEN protein (Fig. 5J).

**Fig. 6:**
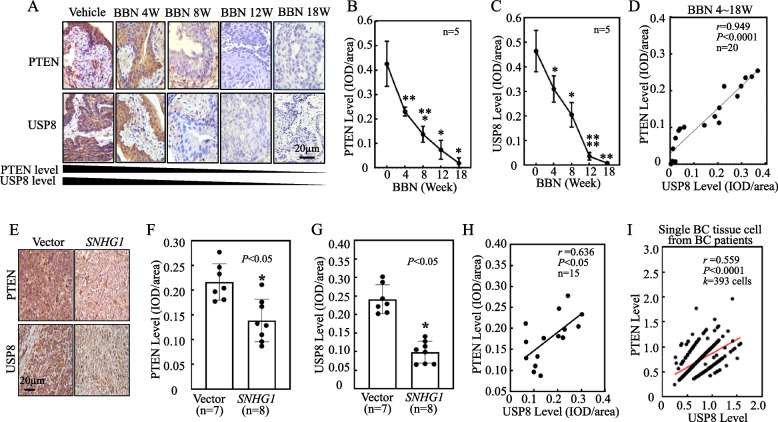
PTEN protein expression exhibits a positive correlation with USP8 expression levels both in vivo and at the single-cell dimension. **A** PTEN and USP8 expression in BBN-treated mouse urothelium were assessed via immunohistochemistry (IHC). **B**-**C** Time-dependent analysis of PTEN and USP8 expression levels in BBN-treated mouse bladder urothelium, denoted by integrated optical density (IOD); * *p* < 0.05, **p < 0.01, ****p < 0.0001. **D** Correlation between PTEN and USP8 expression levels in BBN-treated mouse urothelium over a period ranging from 4 to 18 weeks, measured by the Pearson coefficient (*r*). **E**–**H** PTEN and USP8 expressions were evaluated in tumor tissues from athymic nude mice injected with *SNHG1*-overexpressed cells and corresponding vector scramble transfectants (**E**). Their expression levels were quantified and depicted by IOD/area (**F**-**G**), and the correlation between PTEN and USP8 was calculated using the Pearson coefficient (**H**). Bars represent the mean ± SD for each group. Student’s t-test was employed to determine the p-value, with an asterisk (*) indicating a significant increase compared to Vector transfectants (*p* < 0.05). **I** The correlation between PTEN and USP8 in 393 cells from BC tissues was analyzed (GSE135337, BC single-cell RNA-seq)

In BBN treated mice, we observed a gradual decline in both PTEN and USP8 protein levels over time (Fig. 6A-C). Further, a strong positive correlation was evident between PTEN and USP8 protein expression in the urothelium of these BBN-treated mice (Fig. 6D). Similar trends were observed in the xenograft tumor model: both PTEN and USP8 protein levels were diminished in *SNHG1* overexpressed tumor tissue, and these levels showed a positive correlation (Fig. 6E-H). After analysis of RNA-seq of individual 393 cells from BC tissues, we found that PTEN was positively correlated with USP8 (Fig. 6I). Taken together, we discovered that USP8 binds to PTEN protein, and consequently de-ubiquitinates and stabilizes PTEN protein.

### *SNHG1* promoted *USP8* mRNA degradation through directly interacting with HUR protein

**Fig. 7:**
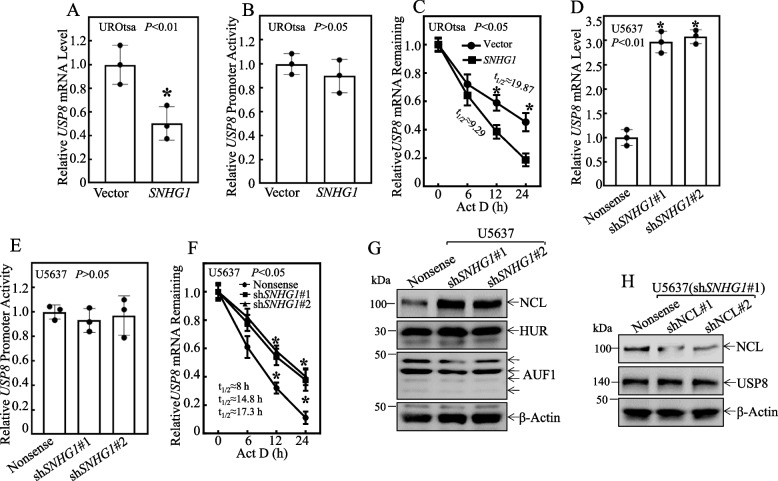
*SNHG1* promotes *USP8* mRNA Degradation. **A** and **D** Total RNA was extracted from UROtsa(*SNHG1*) *versus* UROtsa(Vector) cells, and U5637(sh*SNHG1*#1) cells, U5637(sh*SNHG1*#2) *versus* U5637(Nonsense) cells using Trizol reagent. Real-time PCR assays were conducted to measure *USP8* mRNA expression levels, with GAPDH serving as an internal control. **B** and **E** A luciferase reporter driven by the human USP8 promoter was utilized to assess promoter transcription activity in the indicated transfectants. Results were normalized against internal TK activity. **C** and **F** The specified cells were seeded into 6-well plates and cultured until they reached 80% confluence. Post-synchronization, the cells were treated with Act D for designated durations. Total RNA was subsequently isolated and subjected to real-time PCR analysis to ascertain *USP8* mRNA levels, again using GAPDH as an internal control. **G** Western blot analysis was performed on the chosen cell extracts to determine protein expression levels of NCL, AUF1, and HUR, with β-Actin acting as a protein loading control. **H** An NCL shRNA construct was stably transfected into U5637(sh*SNHG1*#1) cells. The efficiency of NCL protein knockdown and the expression of USP8 were evaluated through Western blotting, utilizing β-Actin as a protein loading control

To elucidate the mechanisms underlying *SNHG1* inhibition of USP8 expression, we first evaluated the potential effects of *SNHG1* on the *USP8* mRNA level and USP8 promoter-driven luciferase reporter activity. As shown in Fig. 7A, *USP8* mRNA level was significantly inhibited in UROtsa(*SNHG1*) in comparison to UROtsa(Vector) cells. Interesting, while *SNHG1* inhibited *USP8* mRNA expression, it did not impact *USP8* promoter-driven luciferase reporter activity (Fig. 7B), thereby ruling out transcriptional regulation of USP8 by *SNHG1*. Subsequently, we investigated the potential effect of *SNHG1* on the stability of *USP8* mRNA. UROtsa cells were treated with Act D over a time course and the results showed that *USP8* mRNA degradation rates in UROtsa(*SNHG1*) cells were much faster than that in UROtsa(Vector) cells (Fig. 7C). These observations indicate that *SNHG1* expedites *USP8* mRNA degradation in UROtsa cells. These findings were also consistent observed in human invasive U5637 cells (Fig. 7D-F).

It is well-documented that mRNA binding proteins such as NCL, AUF1, and HUR can bind to their targeted mRNAs to regulate their stability [43–48]. Thus, we next tested whether these RNAbinding proteins were involved in the *SNHG1*-mediated downregulation of *USP8* mRNA stability. Figure 7G revealed that knockdown of *SNHG1* expression notably increased NCL protein abundance, but had no discernible effect on AUF1 and HUR. To ascertain whether NCL destabilized *USP8* mRNA, thereby regulating USP8 protein expression in U5637(sh*SNHG1*#1) cells, we stably transfected shRNA specific targeting NCL into U5637(sh*SNHG1*#1) cells. Knockdown of NCL expression did not yield any noticeable changes in USP8 protein expression (Fig. 7H), suggesting that NCL was not a *SNHG1* downstream effector mediating its regulation of *USP8* mRNA stability. Given our most recent studies indicating that HUR protein binds to *USP8* mRNA and stabilizes *USP8* mRNA [49], and lncRNAs binds to their targeted protein and affects their bound protein function [50–55], we further probed the potential interaction between *SNHG1* and HUR protein and the data obtained from the online analyses using catRAPID, RPISeq, StarBase V2.0 did suggest their interaction.

**Fig. 8:**
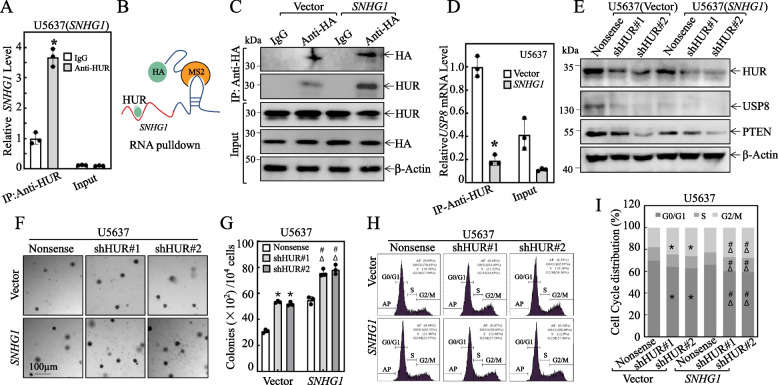
*SNHG1* promotes *USP8* mRNA degradation through direct interaction with HUR protein. **A** The *SNHG1* expression level in anti-HUR pulled-down complex was analyzed using real-time PCR, with Control IgG serving as a reference. The results are depicted as relative *SNHG1* levels, and bars represent the mean ± SD of triplicate measurements. An asterisk (*) indicates a significant increase compared to the complex pulled down with control IgG (*p* < 0.05). **B** A schematic diagram illustrates the binding of *SNHG1* with HUR. **C** U5637 cells were transfected with HA-MS2 in combination with either *SNHG1*-MS2overexpressing or pSL-MS2-12X control constructs. Co-immunoprecipitation was executed using anti-HA antibody-conjugated agarose beads, and the immunoprecipitates were subsequently analyzed by Western blotting to detect HUR. **D** The *USP8* mRNA expression level was assessed using qRT-PCR, with IgG acting as a control. The results are presented as relative *SNHG1* levels, and bars signify the mean ± SD of triplicates. An asterisk (*) denotes a significant difference compared to the vector transfectant (*p* < 0.05). **E** HUR shRNA construct and the nonsense were stably transfected into U5637(Vector) and U5637(*SNHG1*); and the expression of HUR, USP8 and PTEN were evaluated by Western blot. **F**-**G** Anchorage-independent assay was performed for all cell lines as indicated, the representative images were shown (**F**), the number of colonies were counted and compared (**G**). * P < 0.05 *versus* U5637(Vector/Nonsense), #* P* < 0.05 *versus* U5637(*SNHG1*/Nonsense), Δ* P* < 0.05 *versus* U5637(Vector/shHUR). **H**-**I** Cell cycle analysis. The percentages of indicated cell line in different cell cycle stages were tested by flow cytometry, the representative results were showed (**H**), then they were compared by *t*-test (**I**). * P < 0.05 *versus* U5637(Vector/Nonsense), #* P* < 0.05 *versus* U5637(*SNHG1*/Nonsense), Δ* P* < 0.05 *versus* U5637(Vector/shHUR)

To investigate this hypothesis, we conducted RNA-IP assays followed by qRT-PCR tests. Our data confirmed that HUR did specifically bind to *SNHG1* (Fig. 8A). To substantiate this interaction, we further stably co-transfected *SNHG1*-MS2-overexpressing construct with HA-MS2-GFP into U5637 cells and performed the immunoprecipitation (IP) assay to verify the interaction of *SNHG1* with HUR*.* As shown in Fig. 8B and C, HUR was specifically presented in the immune complex pulled down of HA-MS2 by using the anti-HA antibody in transfectants harboring *SNHG1*-MS2overexpressing, corroborating that *SNHG1* does interact with HUR protein. Based on the HUR interaction with *USP8* mRNA or *SNHG1*, we postulated that *SNHG1* might serve as a competitive endogenous RNA for *USP8* mRNA by binding with HUR protein. To examine this notion, we assessed the *USP8* mRNA level precipitated by the anti-HUR antibody in U5637(*SNHG1*) *vs.* U5637(Vector) cells. The results showed that ectopic expression of *SNHG1* dramatically attenuated the *USP8* mRNA level that was precipitated by the anti-HUR antibody in comparison to its scramble vector transfectant (Fig. 8D), suggesting that negative correlation of *SNHG1* expression and *USP8* mRNA expression.

In our quest to conclusively demonstrate that *SNHG1* influence over downstream genes and cellular behaviors hinges on HUR, we had successfully engineered shHUR into U5637(Vector) and U5637(*SNHG1*) for the targeted knockdown of HUR, as illustrated in Fig. 8E. Our findings revealed a significant insight: knocking down HUR in U5637(Vector) contrasted sharply with U5637(Vector/Nonsense), leading to a notable reduction in USP8 and PTEN protein levels (Fig. 8E). This reduction was not just a molecular change; it translated into a pronounced enhancement in the cell anchorage-dependent proliferation abilities, as captured in Fig. 8F and G, and altered the cell cycle dynamics, increasing the proportion of cells in the G2/M phase while decreasing those in the G0/G1 phase (Fig. 8H and I). These results pointed to a critical role of HUR in sustaining USP8 stability. The knockdown of HUR initiated a cascade of cellular changes, starting from a decrease in USP8 and PTEN, leading to escalated cell division and augmented anchorage-dependent proliferation capabilities. In a parallel scenario, knocking down HUR in U5637(*SNHG1*), as compared to U5637(*SNHG1*/Nonsense), yielded similar outcomes, as evidenced in Fig. 8E-I. Importantly, U5637(*SNHG1*/shHUR) stood out, demonstrating an even more potent ability in anchorage-dependent proliferation, with a higher fraction of cells progressing through the S and G2/M phases and fewer remaining in the G0/G1 phase, as detailed in Fig. 8F-I. This finding underscores the fact that HUR knockdown can further amplify the effects of *SNHG1* overexpression on downstream genes and cellular proliferation, solidifying the notion that *SNHG1* regulatory control over these genes and cell behaviors is intricately dependent on HUR.

In summary, these results demonstrate that *SNHG1* can competitively bind to HUR protein, by which reduces its binding with *USP8* mRNA, thereby diminishing HUR’s ability to stabilize USP8 mRNA. This, in turn, accelerates the degradation of PTEN protein, ultimately promoting malignant transformation in urothelial cells and facilitating BMIBC anchorage-independent growth/tumorigenesis.

## Discussion

**Fig. 9:**
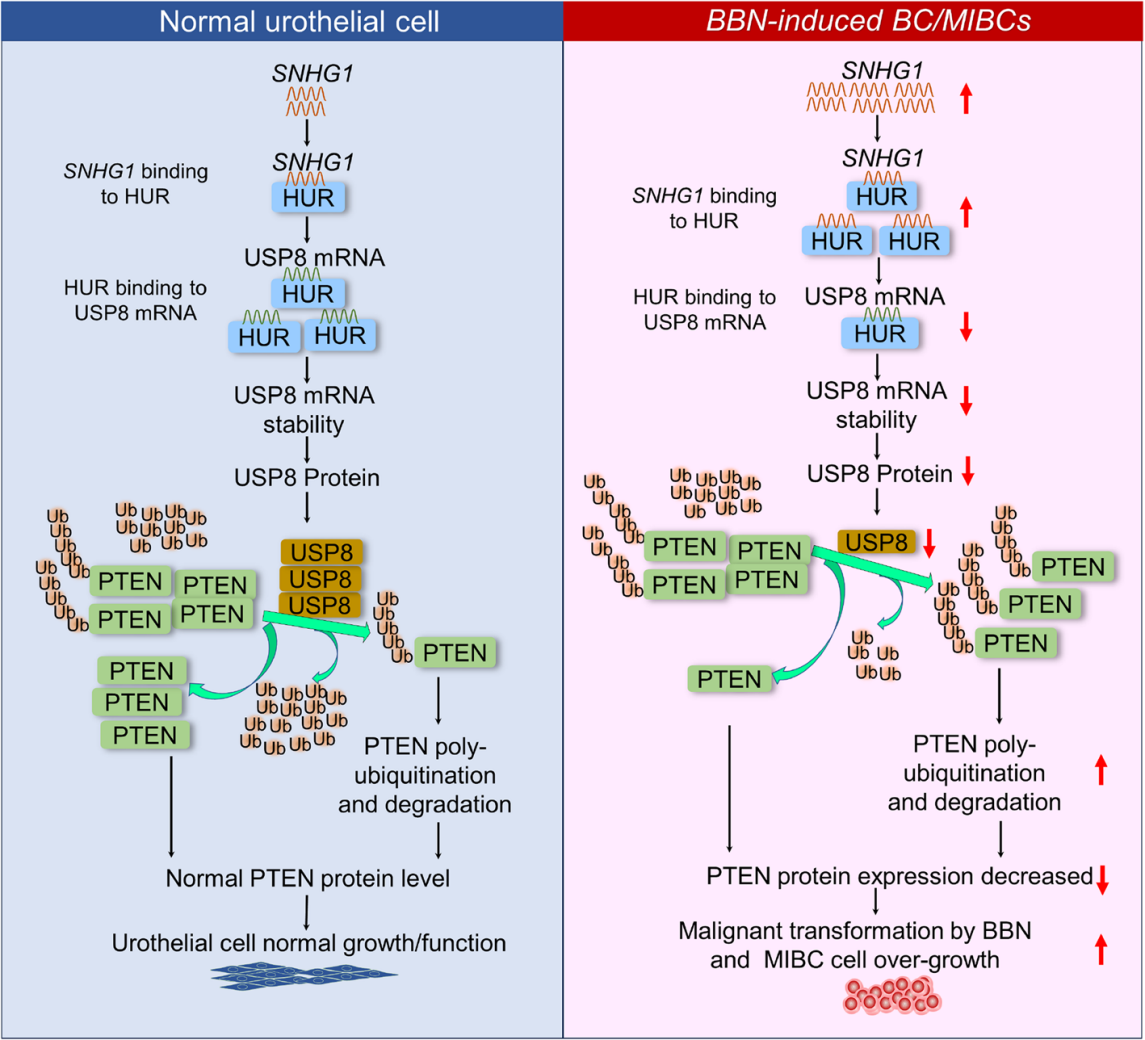
The graphical abstract of this study. It outlines the mechanisms by which *SNHG1* overexpression alone drives normal urothelial cell transformation and fosters anchorage-independent growth in human BMIBC cells through downregulating PTEN protein

BC stands as the leading cause of death among malignancies of the urinary system globally [56]. Despite surgical interventions like radical cystectomy, an alarming 50–60% of MIBCs progress to metastatic stages, accounting for nearly all BC-related fatalities [57–60]. Consequently, understanding the underlying biological mechanisms is paramount for effective disease management. Our research identifies *SNHG1*, a lncRNA, as a pivotal regulator in the transformation of human urothelial cells and the promotion of tumorigenesis in basal muscle invasive BC cells. In mouse models exposed to BBN, a bladder-specific carcinogen, we observed the initiation of primary BMIBCs alongside significant downregulation of the PTEN tumor-suppressor protein. Intriguingly, *SNHG1* was markedly upregulated in a time-dependent manner in normal human urothelial cells (UROtsa), and its ectopic expression was sufficient to induce malignant transformation, suggesting *SNHG1*’s potential to mimic the carcinogenic effects of BBN. Functional studies employing both overexpression and knockdown approaches further substantiated *SNHG1*’s critical role in enabling anchorage-independent growth and tumorigenesis in human BMIBCs. Our mechanistic insights revealed that *SNHG1* directly interacts with the mRNA binding protein HUR. This interaction leads to a reduction in HUR’s affinity for USP8 mRNA, triggering its degradation and subsequently inducing PTEN protein polyubiquitination and degradation. This cascade of events ultimately drives both urothelial cell transformation and BC cell tumorigenesis, as illustrated in Fig. 9. These findings not only deepen our understanding of BC pathogenesis but also spotlight *SNHG1* as a potential key player, opening avenues for new diagnostic and therapeutic strategies.

Elevated expression of the RNA *SNHG1* has been implicated in various forms of cancer [18, 19]. Specifically, its high expression levels in colorectal cancer (CRC) tissues have been linked to both metastasis and unfavorable patient outcomes. This is predominantly due to the activation of the Wnt/βcatenin pathway [61] and the subsequent upregulation of key proteins such as β-catenin and MMP-9 [62]. Furthermore, the oncogenic impact of *SNHG1* on CRC cell proliferation is connected to its ability to suppress the p53 tumor suppressor gene [63] and to sequester miR-145 [64]. In addition, *SNHG1* fosters cancer cell growth by inhibiting miR-195 and miR-199a, while also facilitating cancer invasion through its direct interaction with the PP2A catalytic subunit, ultimately triggering autophagy [20, 65, 66]. In our recent research, we have uncovered a significant finding: *SNHG1* is markedly upregulated in both human urothelial cells in vitro and mouse urothelium in vivo when exposed to the chemical carcinogen BBN, showing a time-dependent increase in expression levels. More importantly, we have found that the mere overexpression of *SNHG1* is sufficient to initiate malignant transformation in human urothelial cells. This upregulation plays a vital role in enhancing both anchorage-independent cell growth in vitro and tumorigenicity in nude mice in vivo. Mechanistically, we found that this involves the degradation of the tumor suppressor protein PTEN in invasive bladder cancer cells. This new insight not only expands our understanding of *SNHG1*’s multifaceted role in oncogenesis but also highlights its potential as a key molecular player in urothelial malignancies.

PTEN is a well-established tumor suppressor gene, commonly subject to mutations or downregulation across various cancer types, including bladder cancer. It has been demonstrated in earlier research that PTEN functions as a tumor suppressor by negatively modulating the PI3K/Akt/mTOR signaling pathway [12, 15, 67]. Recent studies have also highlighted the influence of lncRNAs on PTEN expression. For instance, a study by Ma et al. revealed that the anti-differentiation noncoding RNA (ANCR) facilitates cell proliferation and confers radiation resistance in nasopharyngeal carcinoma by suppressing PTEN transcription. This occurs through ANCR’s interaction with the oncogenic histone methyltransferase component of the polycomb repressive complex 2 [68]. In our current work, we discovered that the overexpression of *SNHG1* enables it to bind with the mRNA-binding protein HUR. This interaction interferes with HUR’s ability to stabilize USP8 mRNA, leading to its degradation. The subsequent reduction in USP8 levels triggers the degradation and downregulation of PTEN protein. This cascade of molecular events results in the transformation of UROtsa cells and promotes anchorage-independent growth in human invasive U5637 cells, further underscoring the intricate regulatory networks that influence cancer progression. Ubiquitination involves the covalent attachment of poly-ubiquitin chains to target proteins, facilitated through a series of signaling events. Deubiquitinating enzymes (DUBs) are specialized proteases that can undo this process by modifying or disassembling these polyubiquitin chains [69]. This dynamic equilibrium between ubiquitination and deubiquitination plays a pivotal role in numerous cellular functions and regulatory mechanisms [70]. Prior research has indicated that the degradation of the PTEN protein is controlled by the ubiquitin-dependent proteasomal degradation pathway [10, 71], with E3 ligases such as NEDD4, WWP2, and XIAP acting as mediators for PTEN ubiquitination [13]. Our current investigation unveils that *SNHG1* accelerates the degradation of PTEN protein via this ubiquitin-dependent pathway. Interestingly, *SNHG1*’s influence did not significantly alter the levels of the afore mentioned E3 ligases. Additionally, we observed that overexpression of *SNHG1* effectively suppressed USP8 levels, while its knockdown led to a substantial increase in USP8 expression. As DUBs can serve as either inhibitors or activators of the ubiquitin system, they play a crucial role in regulating protein degradation [72, 73]. USP8, an endosome-associated DUB, has been found to control the ubiquitination and degradation of various membrane proteins [74, 75]. Earlier studies demonstrated that USP8 serves as a novel deubiquitylase for the Notch1 intracellular domain, influencing the Notch signaling pathway. Our gain-and-loss functional assays further showed that USP8 positively regulates PTEN protein levels. Notably, USP8 directly interacts with PTEN, reducing its ubiquitination. These groundbreaking findings reveal PTEN as a novel substrate for USP8, and indicate that PTEN degradation facilitated by *SNHG1* overexpression is directly mediated through the deubiquitin-specific enzyme USP8. The abnormal expression of *SNHG1* and PTEN was also confirmed in TCGA database. We found that *SNHG1* was significantly up-regulated in human bladder cancer tissues, and PTEN protein level was significantly downregulated in advanced bladder cancer tissues (Figure S1), which supported our conclusion. To summarize, our research reveals that elevated *SNHG1* levels significantly contribute to the transformation of normal human urothelial cells and to the anchorage-independent growth and tumorigenesis of human BMIBC. This occurs primarily through the suppression of PTEN protein expression. The *SNHG1*-induced decline in PTEN is attributable to an increase in PTEN protein degradation, facilitated by the reduction of USP8dependent deubiquitination. Moreover, *SNHG1* disrupts the stability of USP8 mRNA by competing with it for binding to the HUR protein. The regulatory axis we've identified, involving *SNHG1* and PTEN in the onset and progression of BC, holds potential for future diagnostic and therapeutic strategies. It was important to note that in our study, the *SNHG1*-PTEN axis demonstrated a strong correlation relative to other signaling proteins we investigated. We acknowledge that the pathways influenced by PTEN are likely to be multifaceted, and *SNHG1* may not be the sole regulator. Looking ahead, we will employ omics techniques to identify key gene sets that are significantly altered within the PTEN signaling pathway.

## Conclusion

Our work unveils a groundbreaking insight: PTEN is a novel substrate for USP8. Overexpressed *SNHG1* competes with USP8 for binding to HUR, leading to decreased USP8 mRNA stability and protein expression. This chain of events culminates in PTEN degradation and consequently fosters both urothelial transformation and the anchorage-independent growth of invasive BC cells. Alongside our recent findings that *SNHG1* promotes BC invasion and metastasis [20], these discoveries underscore the capability of *SNHG1* to emulate the carcinogenic effects of the bladder-specific chemical carcinogen BBN. The implications of these findings are substantial, offering new avenues for understanding the pivotal role of *SNHG1* in the promotion and progression of BC. Not only does this position *SNHG1* or PTEN as a promising new biomarker for the early detection, diagnosis, and prognostic assessment of BC, but it also suggests that targeting *SNHG1* or PTEN can be a viable strategy for the prevention and treatment of BCs.

### Supplementary Information

**Additional file 1: Table S1.**The high-throughput sequencing results for mouse urothelium from BBN-treated mouse at different time points (w, week).

**Additional file 2: Figure S1.** The level of *SNHG1* or PTEN in bladder cancer based on TCGA database. (A-B) Comparison of the levels of *SNHG1* in patients diagnosed with bladder cancer *vs* the normal by paired (A) or unpaired test (B). (C-D) PTEN protein levels in different T (C) or M (D) categories. BLCA, Bladder Urothelial Carcinoma. *p<0.05, ***p<0.001, ****p<0.0001. **Figure S2.** The level of *SNHG1* in normal human bladder urothelial cell and human bladder cancer cell lines. *p<0.05. **Figure S3.** Athymic nude mice received subcutaneous injections of either *SNHG1*-overexpressing T24T cells or their corresponding vector scramble controls (5×10^6^ cells suspended in 100 *μ*L PBS) into the right axillary region. Six weeks post-injection, mice were photographed (A), and subsequently, tumors were surgically excised for analysis. Immunohistochemical staining for Ki67 was performed on the excised tumor tissues (B-C). Data are presented as mean ± SD for each group. Statistical significance was determined using Student’s *t*-test, with an asterisk (*) denoting a significant increase relative to the vector control group (*p* < 0.05). **Figure S4.** The figure presents representative microscopic images illustrating the results of the anchorage-independent growth assay across various cell lines. Notably, images for UROtsa cells either overexpressing *SNHG1* or with vector control are shown in (A). Similarly, U5637 cells with *SNHG1* overexpression or vector control are displayed in (B), and T24T cells with *SNHG1* overexpression or vector control are depicted in (C). Additionally, images of U5637 cells with targeted *SNHG1* knockdown (sh*SNHG1*#1 and sh*SNHG1*#2) alongside a nonsense control are provided in (D). Corresponding images for T24T cells subjected to *SNHG1 *knockdown (sh*SNHG1*#1 and sh*SNHG1*#2) and a nonsense control are displayed in (E). All images were captured after a three-week incubation period. **Figure S5.** *SNHG1* promoted the cell cycle progression of human normal bladder urothelial cell and bladder cancer cell line. (A-D) Effect of *SNHG1* overexpression on cell cycle progression in UROtsa (A-B) and T24T (C-D) detected by flow cytometry. (E-F) Comparison of the cell cycle progression of T24T (sh*SNHG1*) *vs* T24T (Nonsense) examined by flow cytometry. *p<0.05. **Figure S6. **Downregulation of PTEN mediated *SNHG1’*s promotion of anchorage cell growth of human BC cell line T24T. (A) A PTEN-overexpressing construct and its vector were stably transfected into T24T (*SNHG1*) or T24T (Vector) cells, and Western blot analysis was performed to confirm PTEN protein expression. (B-C) Representative images of the colonies of anchorage-independent assay (B) and their statistical analysis (C) for cell lines in (A). (D) PTEN shRNA construct and the corresponding nonsense were stably transfected into T24T (sh*SNHG1*) or T24T (Nonsense) cells, and transfection efficiency was performed to confirm PTEN protein expression. (E-F) The colonies of anchorage-independent assay were captured under microscopy (E), the number of colonies per 10,000 cells for cell lines in (D) were compared (F). *p<0.05. **Figure S7. **Effect of USP8 on *SNHG1’*s promotion of anchorage cell growth of human BC cell line. (A) USP8 shRNA construct and the nonsense were stably transfected into T24T (sh*SNHG1*) or T24T (Nonsense) cells and identified by Western blot. (B-C) We did anchorage-independent assay for all cell lines in (A), the colonies representative images captured by microscope (B), the colony count was conducted, with results presented as colonies per 10,000 cells from three separate experiments (C). *p<0.05.

## Data Availability

The data that support the findings of this study are available from the corresponding author or the first author upon reasonable request.

## Abbreviations

PTEN: Phosphatase and tensin homolog deleted on chromosome ten
BC: Bladder cancer
BBN: N-butyl-N-(4-hydroxybu-tyl) nitrosamine
BMIBC: Basal muscle invasive bladder cancer
*SNHG1*: Small nucleolar RNA host gene 1
USP8: Ubiquitin-specific peptidase 8
MIBC: Muscle invasive bladder cancer
lncRNA: Long noncoding RNA
sh*SNHG1*: ShRNA constructs specifically targeting human *SNHG1*
NCL: Nucleolin
HUR: ELAV like RNA binding protein 1, also known as ELVAL1
Act D: Actinomycin D
CHX: Cyclohexamide
FBS: Fetal bovine serum
RIP: RNA Immunoprecipitation
PI: Propidium Iodide
IHC: Immunohistochemistry Staining
TCGA: The Cancer Genome Atlas
DUBs: Deubiquitinating enzymes
CRC: Colorectal cancer
ANCR: Anti-differentiation noncoding RNA
