# Supplementary material for: Tumorigenesis of basal muscle invasive bladder cancer was mediated by PTEN protein degradation resulting from SNHG1 upregulation
Source: J Exp Clin Cancer Res. 2024 Feb 17;43:50. doi: 10.1186/s13046-024-02966-4 (PMC10874020; doi:10.1186/s13046-024-02966-4)
Supplement: Supplementary file 2 — Additional file 2: Figure S1. The level of SNHG1 or PTEN in bladder cancer based on TCGA database. (A-B) Comparison of the levels of SNHG1 in patients diagnosed with bladder cancer vs the normal by paired (A) or unpaired test (B). (C-D) PTEN protein levels in different T (C) or M (D) categories. BLCA, Bladder Urothelial Carcinoma. *p<0.05, ***p<0.001, ****p<0.0001. Figure S2. The level of SNHG1 in normal human bladder urothelial cell and human bladder cancer cell lines. *p<0.05. Figure S3. Athymic nude mice received subcutaneous injections of either SNHG1-overexpressing T24T cells or their corresponding vector scramble controls (5×10^6^ cells suspended in 100 μL PBS) into the right axillary region. Six weeks post-injection, mice were photographed (A), and subsequently, tumors were surgically excised for analysis. Immunohistochemical staining for Ki67 was performed on the excised tumor tissues (B-C). Data are presented as mean ± SD for each group. Statistical significance was determined using Student’s t-test, with an asterisk (*) denoting a significant increase relative to the vector control group (p < 0.05). Figure S4. The figure presents representative microscopic images illustrating the results of the anchorage-independent growth assay across various cell lines. Notably, images for UROtsa cells either overexpressing SNHG1 or with vector control are shown in (A). Similarly, U5637 cells with SNHG1 overexpression or vector control are displayed in (B), and T24T cells with SNHG1 overexpression or vector control are depicted in (C). Additionally, images of U5637 cells with targeted SNHG1 knockdown (shSNHG1#1 and shSNHG1#2) alongside a nonsense control are provided in (D). Corresponding images for T24T cells subjected to SNHG1 knockdown (shSNHG1#1 and shSNHG1#2) and a nonsense control are displayed in (E). All images were captured after a three-week incubation period. Figure S5. SNHG1 promoted the cell cycle progression of human normal bladder urothelial cell and bladder [file 13046_2024_2966_MOESM2_ESM.pptx]

## Slide 1
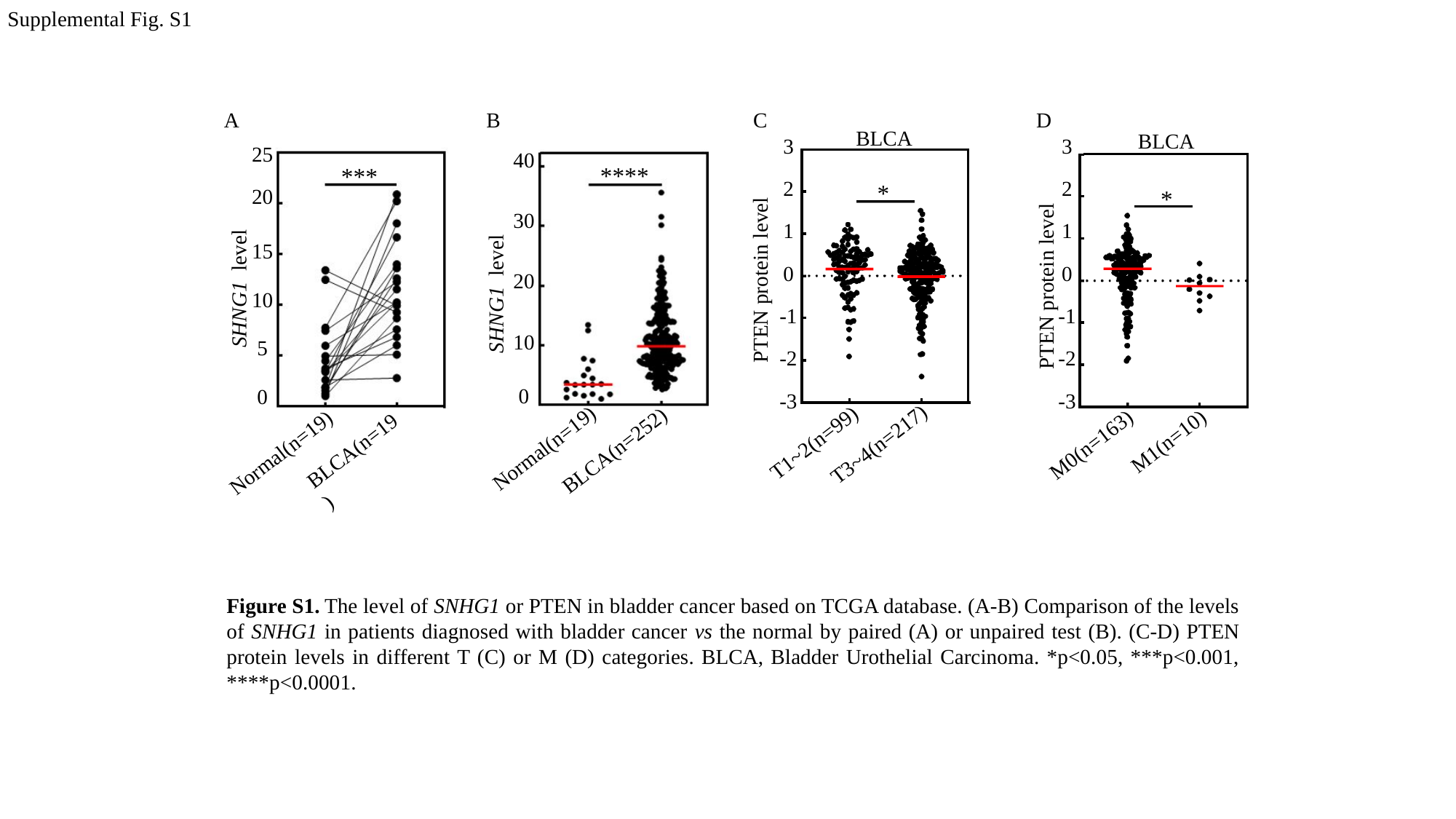

Supplemental Fig. S1
3
2
1
0
-1
-2
-3
3
2
1
0
-1
-2
-3
A
B
C
D
40
30
20
10
0
BLCA
BLCA
25
20
15
10
5
0
****
***
*
*
SHNG1 level
PTEN protein level
PTEN protein level
SHNG1 level
M1(n=10)
T3~4(n=217)
BLCA(n=252)
T1~2(n=99)
M0(n=163)
Normal(n=19)
BLCA(n=19)
Normal(n=19)
Figure S1. The level of SNHG1 or PTEN in bladder cancer based on TCGA database. (A-B) Comparison of the levels of SNHG1 in patients diagnosed with bladder cancer vs the normal by paired (A) or unpaired test (B). (C-D) PTEN protein levels in different T (C) or M (D) categories. BLCA, Bladder Urothelial Carcinoma. *p<0.05, ***p<0.001, ****p<0.0001.

## Slide 2
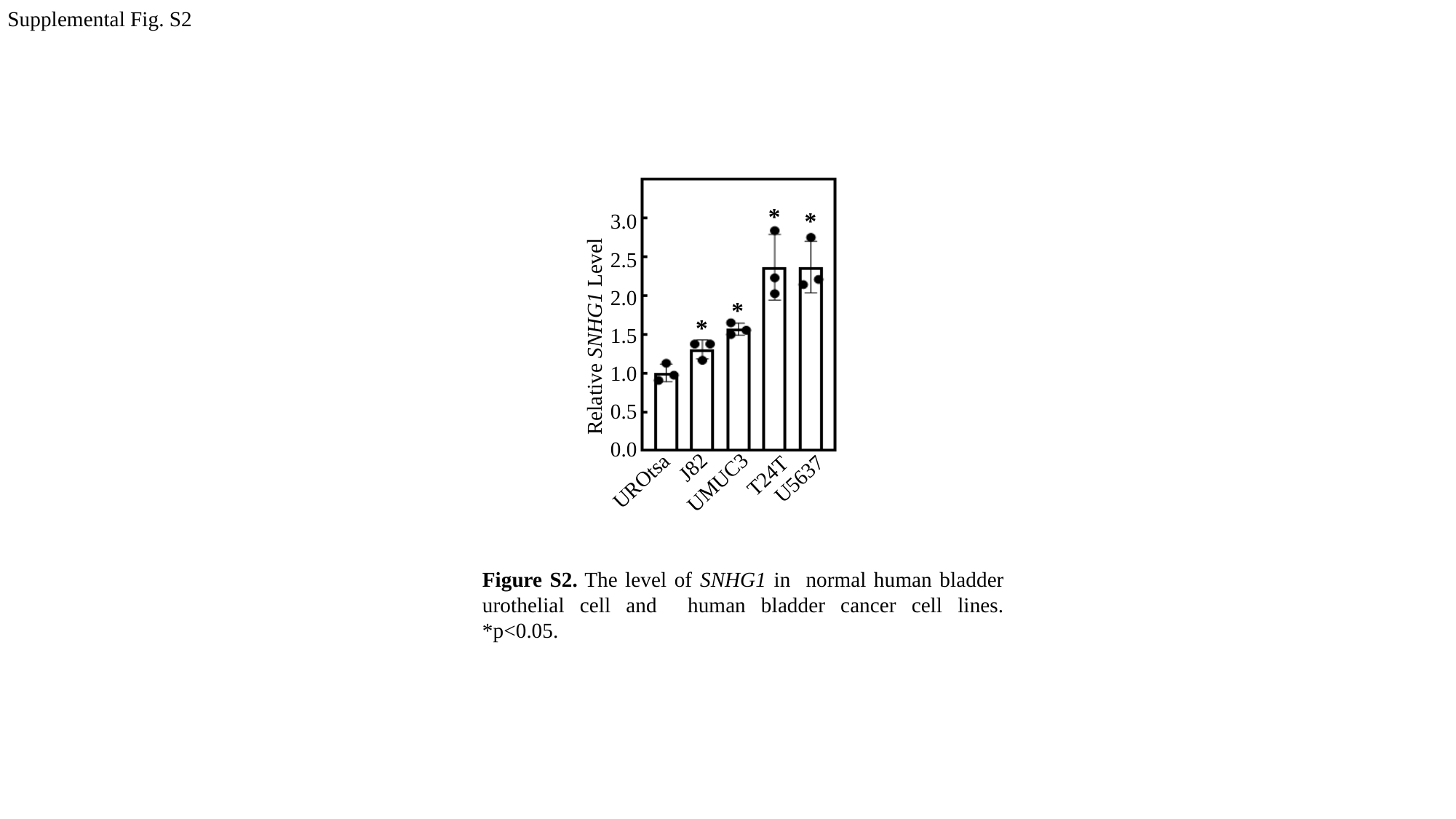

Supplemental Fig. S2
3.0
2.5
2.0
1.5
1.0
0.5
0.0
*
*
*
*
Relative SNHG1 Level
J82
T24T
UMUC3
U5637
UROtsa
Figure S2. The level of SNHG1 in normal human bladder urothelial cell and human bladder cancer cell lines. *p<0.05.

## Slide 3
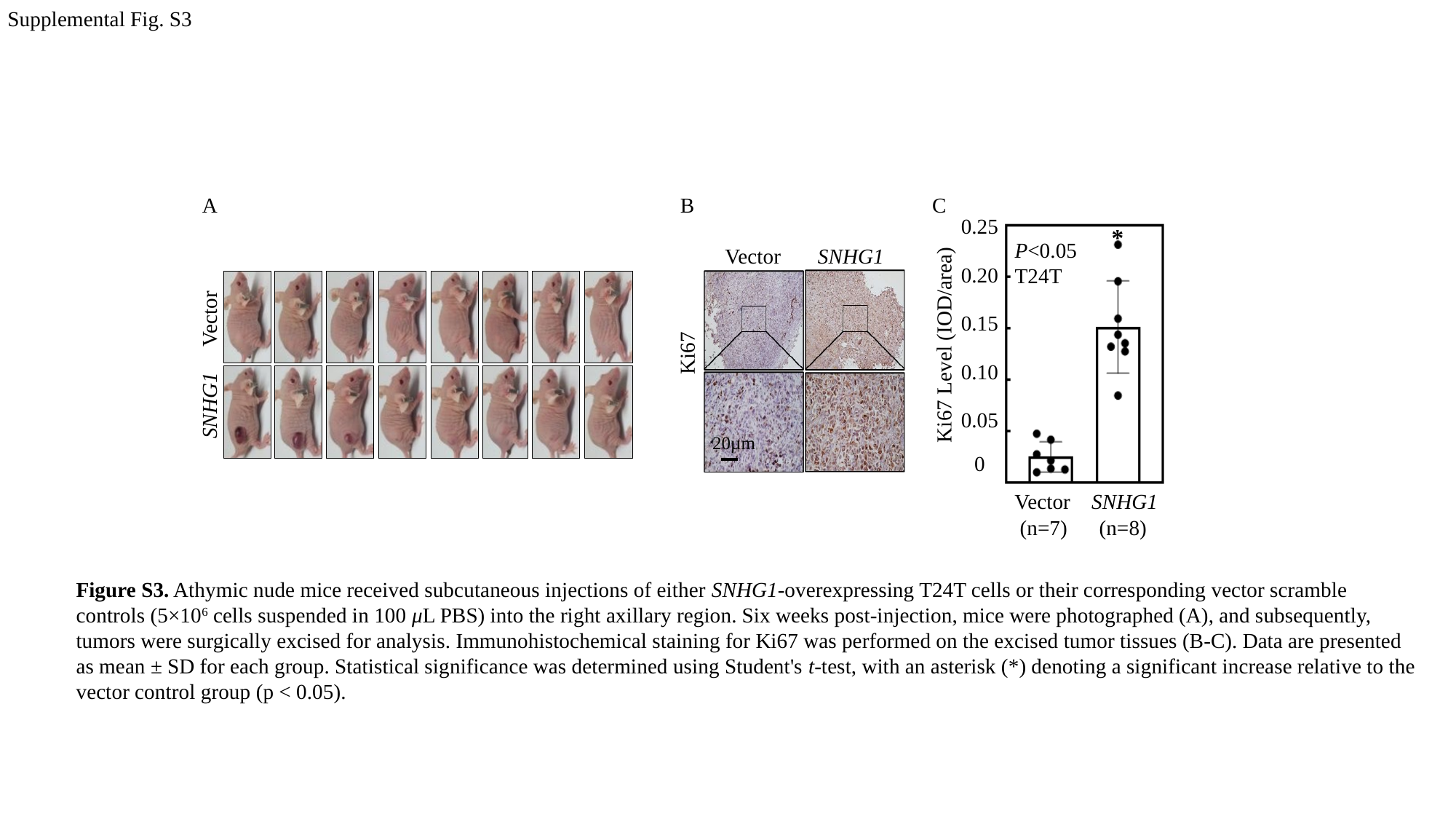

Supplemental Fig. S3
A
B
C
0.25
0.20
0.15
0.10
0.05
0
*
P<0.05
T24T
Vector SNHG1
Ki67 Level (IOD/area)
Ki67
SNHG1 Vector
20µm
Vector SNHG1
 (n=7) (n=8)
Figure S3. Athymic nude mice received subcutaneous injections of either SNHG1-overexpressing T24T cells or their corresponding vector scramble controls (5×10^6^ cells suspended in 100 μL PBS) into the right axillary region. Six weeks post-injection, mice were photographed (A), and subsequently, tumors were surgically excised for analysis. Immunohistochemical staining for Ki67 was performed on the excised tumor tissues (B-C). Data are presented as mean ± SD for each group. Statistical significance was determined using Student's t-test, with an asterisk (*) denoting a significant increase relative to the vector control group (p < 0.05).

## Slide 4
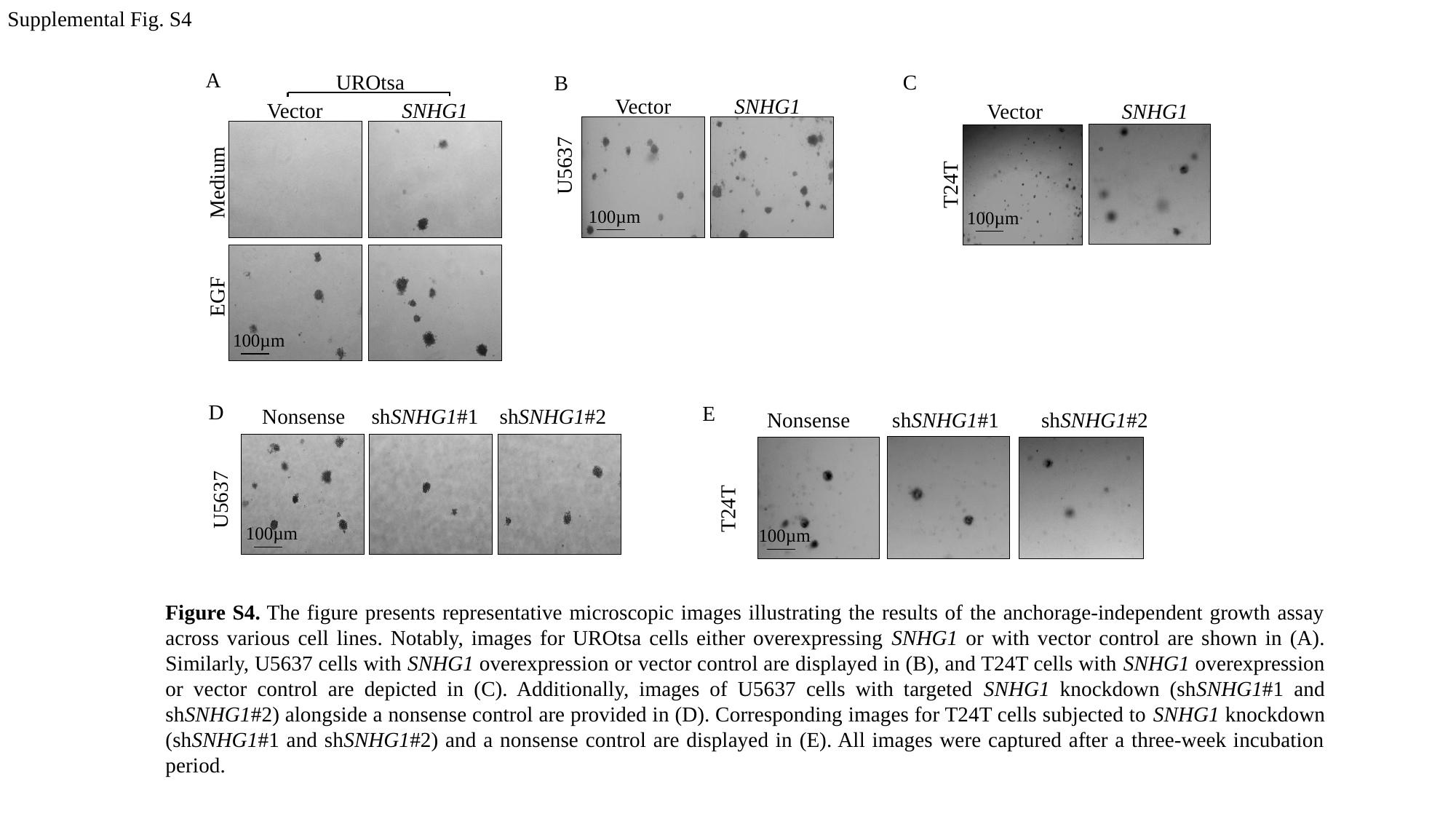

Supplemental Fig. S4
A
UROtsa
C
B
Vector SNHG1
Vector SNHG1
Vector SNHG1
U5637
T24T
EGF Medium
100µm
100µm
100µm
D
E
Nonsense shSNHG1#1 shSNHG1#2
Nonsense shSNHG1#1 shSNHG1#2
U5637
T24T
100µm
100µm
Figure S4. The figure presents representative microscopic images illustrating the results of the anchorage-independent growth assay across various cell lines. Notably, images for UROtsa cells either overexpressing SNHG1 or with vector control are shown in (A). Similarly, U5637 cells with SNHG1 overexpression or vector control are displayed in (B), and T24T cells with SNHG1 overexpression or vector control are depicted in (C). Additionally, images of U5637 cells with targeted SNHG1 knockdown (shSNHG1#1 and shSNHG1#2) alongside a nonsense control are provided in (D). Corresponding images for T24T cells subjected to SNHG1 knockdown (shSNHG1#1 and shSNHG1#2) and a nonsense control are displayed in (E). All images were captured after a three-week incubation period.

## Slide 5
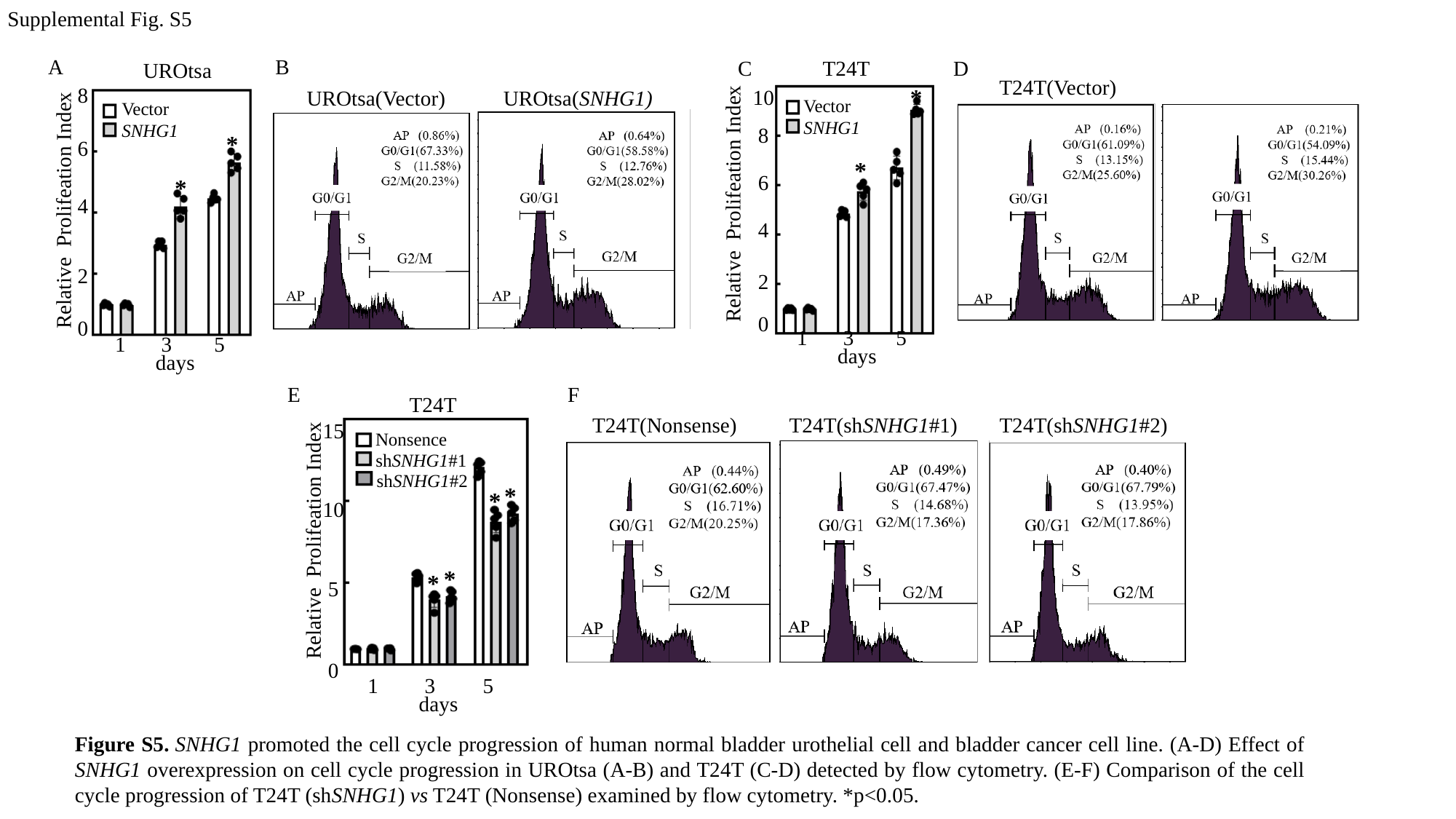

Supplemental Fig. S5
A
B
C
T24T
D
UROtsa
T24T(Vector) T24T(SNHG1)
 8
 6
 4
 2
 0
*
10
 8
 6
 4
 2
 0
UROtsa(Vector) UROtsa(SNHG1)
Vector
SNHG1
Vector
SNHG1
*
*
*
Relative Prolifeation Index
Relative Prolifeation Index
 3 5
 days
 3 5
 days
E
F
T24T
T24T(Nonsense) T24T(shSNHG1#1) T24T(shSNHG1#2)
15
 10
 5
 0
Nonsence
shSNHG1#1
shSNHG1#2
*
*
Relative Prolifeation Index
*
*
 3 5
 days
Figure S5. SNHG1 promoted the cell cycle progression of human normal bladder urothelial cell and bladder cancer cell line. (A-D) Effect of SNHG1 overexpression on cell cycle progression in UROtsa (A-B) and T24T (C-D) detected by flow cytometry. (E-F) Comparison of the cell cycle progression of T24T (shSNHG1) vs T24T (Nonsense) examined by flow cytometry. *p<0.05.

## Slide 6
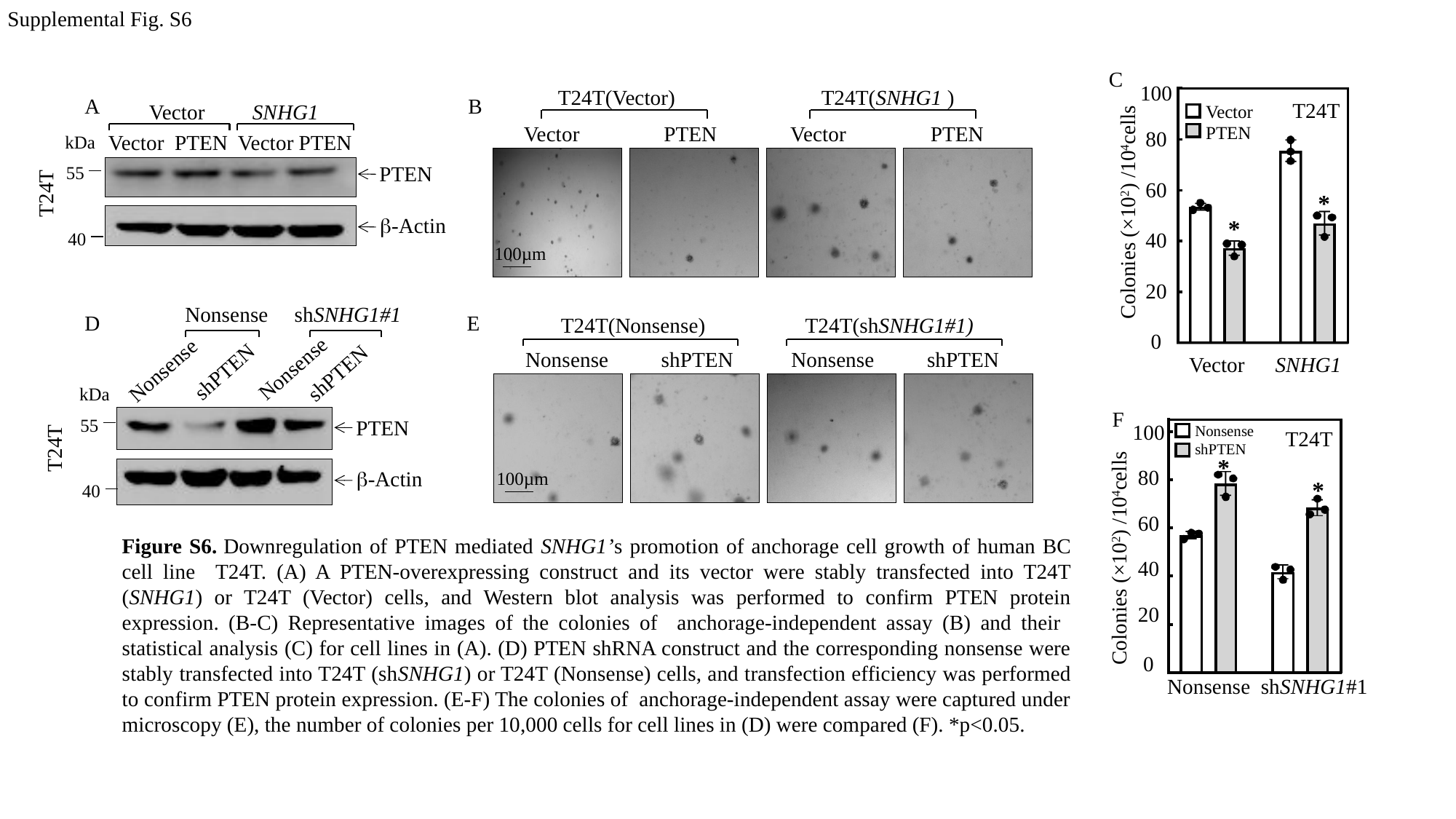

Supplemental Fig. S6
C
100
 80
 60
 40
 20
 0
T24T(Vector)
T24T(SNHG1 )
A
B
Colonies (×102) /104cells
T24T
Vector SNHG1
Vector
PTEN
Vector PTEN Vector PTEN
 Vector PTEN Vector PTEN
kDa
PTEN
55
T24T
*
β-Actin
*
40
100µm
Nonsense shSNHG1#1
D
E
T24T(Nonsense)
T24T(shSNHG1#1)
shPTEN
Nonsense shPTEN Nonsense shPTEN
shPTEN
Vector
SNHG1
Nonsense
Nonsense
kDa
F
55
PTEN
Nonsense
shPTEN
100
 80
 60
 40
 20
 0
T24T
T24T
Colonies (×102) /104cells
*
β-Actin
100µm
*
40
Figure S6. Downregulation of PTEN mediated SNHG1’s promotion of anchorage cell growth of human BC cell line T24T. (A) A PTEN-overexpressing construct and its vector were stably transfected into T24T (SNHG1) or T24T (Vector) cells, and Western blot analysis was performed to confirm PTEN protein expression. (B-C) Representative images of the colonies of anchorage-independent assay (B) and their statistical analysis (C) for cell lines in (A). (D) PTEN shRNA construct and the corresponding nonsense were stably transfected into T24T (shSNHG1) or T24T (Nonsense) cells, and transfection efficiency was performed to confirm PTEN protein expression. (E-F) The colonies of anchorage-independent assay were captured under microscopy (E), the number of colonies per 10,000 cells for cell lines in (D) were compared (F). *p<0.05.
Nonsense shSNHG1#1

## Slide 7
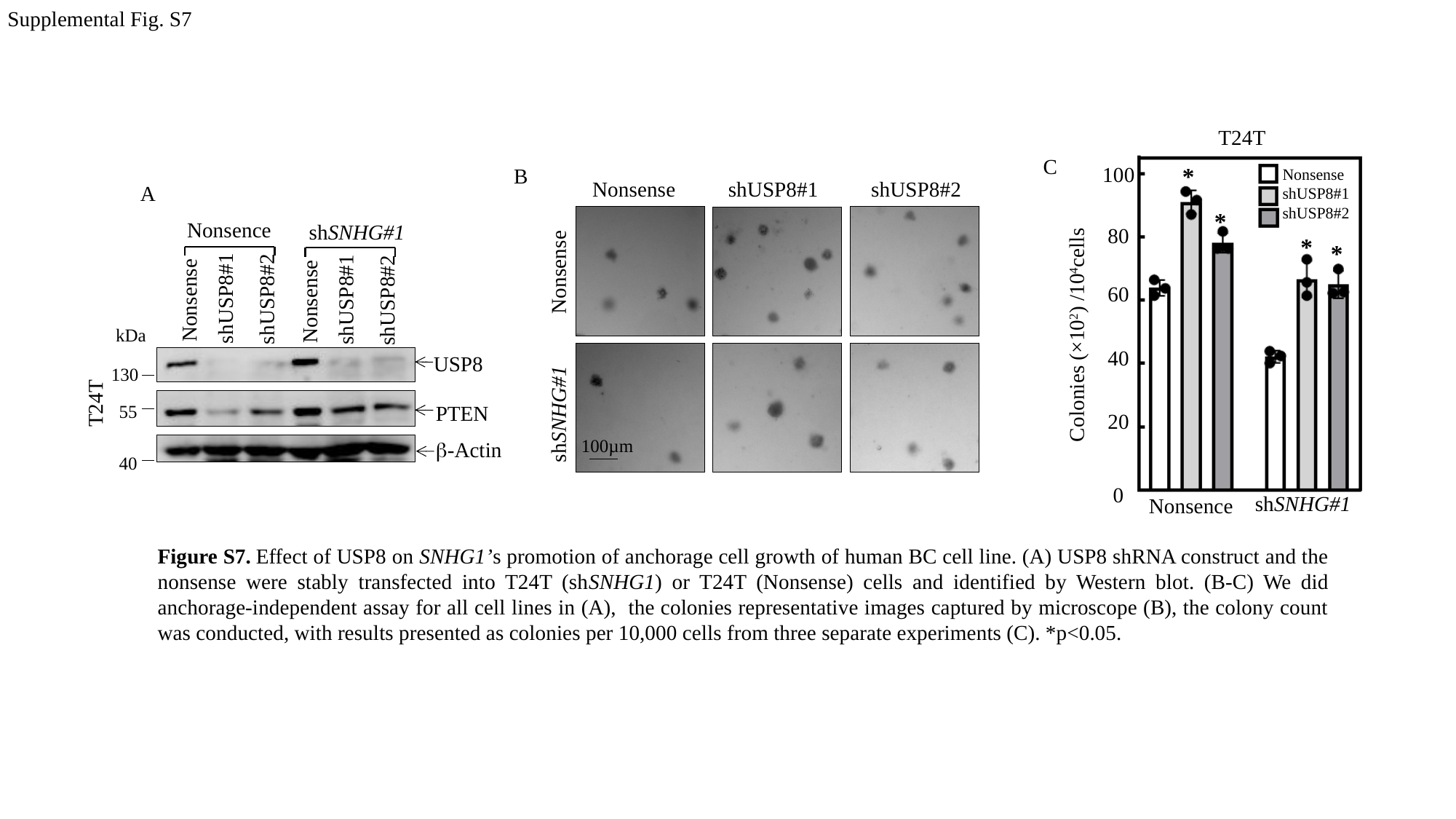

Supplemental Fig. S7
T24T
C
*
 100
 80
 60
 40
 20
 0
B
Nonsense
shUSP8#1
shUSP8#2
Nonsense shUSP8#1 shUSP8#2
A
*
Nonsence
shSNHG#1
Colonies (×102) /104cells
*
*
Nonsense
shUSP8#1
shUSP8#2
shUSP8#1
shUSP8#2
Nonsense
Nonsense
kDa
USP8
130
T24T
PTEN
55
shSNHG#1
100µm
β-Actin
40
shSNHG#1
Nonsence
Figure S7. Effect of USP8 on SNHG1’s promotion of anchorage cell growth of human BC cell line. (A) USP8 shRNA construct and the nonsense were stably transfected into T24T (shSNHG1) or T24T (Nonsense) cells and identified by Western blot. (B-C) We did anchorage-independent assay for all cell lines in (A), the colonies representative images captured by microscope (B), the colony count was conducted, with results presented as colonies per 10,000 cells from three separate experiments (C). *p<0.05.
